# *In situ* electrochemical quantification of active sites in Fe–N/C non-precious metal catalysts

**DOI:** 10.1038/ncomms13285

**Published:** 2016-10-31

**Authors:** Daniel Malko, Anthony Kucernak, Thiago Lopes

**Affiliations:** 1 Department of Chemistry, Imperial College London, South Kensington Campus, London SW7 2AZ, UK; 2 Fuel Cells and Hydrogen Centre, Nuclear and Energy Research Institute, IPEN-CNEN/SP, Sao Paulo 05508-000, Brazil

## Abstract

The economic viability of low temperature fuel cells as clean energy devices is enhanced by the development of inexpensive oxygen reduction reaction catalysts. Heat treated iron and nitrogen containing carbon based materials (Fe–N/C) have shown potential to replace expensive precious metals. Although significant improvements have recently been made, their activity and durability is still unsatisfactory. The further development and a rational design of these materials has stalled due to the lack of an *in situ* methodology to easily probe and quantify the active site. Here we demonstrate a protocol that allows the quantification of active centres, which operate under acidic conditions, by means of nitrite adsorption followed by reductive stripping, and show direct correlation to the catalytic activity. The method is demonstrated for two differently prepared materials. This approach may allow researchers to easily assess the active site density and turnover frequency of Fe–N/C catalysts.

## Abstract (web summary)

Fe-N/C catalysts show surprising activity towards oxygen reduction in fuel cells. However, there is significant uncertainty as to the structure of the active site. Here, the authors quantify the number and turnover frequency of the active sites by reversibly blocking those sites with nitrite (NO_2_^¯^).

## Graphical abstract

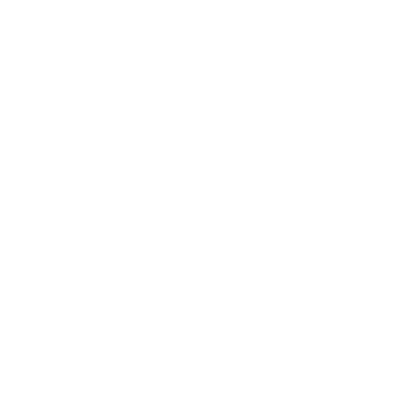

Non-precious metal based oxygen reduction reaction (ORR) catalysts have attracted significant attention as low cost alternatives to Pt-based materials for the use in polymer electrolyte fuel cells^1^,^2^. Especially for heat treated iron, nitrogen and carbon containing materials (Fe–N/C), high activities have been demonstrated^3^,^4^,^5^,^6^. However, these materials need to be improved to be applied in commercial devices. The exact nature of the active site remains elusive. Recent spectroscopic studies provide evidence that one type of highly active sites might be atomic iron centres, possibly coordinated with nitrogen^7^,^8^,^9^. These might chemically behave similarly to haem-like complexes^7^,^8^,^9^. *Ex situ* quantification of iron sites in these materials has recently been demonstrated^10^. However, techniques such as Mössbauer and X-ray absorption spectroscopy are not surface specific, do not allow the direct correlation to the oxygen reduction activity and are impractical^11^. The intrinsic catalytic activity of an electrocatalyst at a given potential is correlated with the current density by the following relationship:


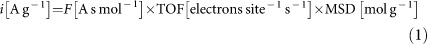


where *i* is the gravimetric current density at a given potential, *F* is Faraday's constant, TOF is the turnover frequency in electrons per site per second and MSD the gravimetric active site density^12^. There are two ways to improve the catalyst performance. Either the turnover frequency or the accessible site density has to be increased^12^. It is therefore necessary to accurately determine these values to develop a rational structure-activity relationship. To date there is no coherent and simple method to measure the intrinsic activity of Fe–N/C catalysts. These materials are usually prepared by high temperature heat treatment of a suitable precursor material that contains carbon, nitrogen and iron^4^,^5^,^13^,^14^. Recently, it has been shown that different routes lead to a similar set of iron sites^11^,^15^,^16^. A common probe to these iron sites would allow a consistent approach to evaluating their activity. Although extensive efforts have been employed, to date no true understanding could be established as how to increase the active site density or turnover frequency. Although changes to catalyst synthesis can lead to changes in electrocatalytic performance, the only clear trend identified to date within Fe–N/C catalysts is a correlation of iron content and activity, indicating that an increase in metallic centres increases the active site density while preserving the TOF^17^. However, for other changes such as different gas treatments, it is not clear whether the increase in activity is due to changes in the activity of the catalyst site (TOF), or the site density (MSD). The latter may occur by improving access to sites through morphological changes in the catalyst structure during treatment. We cannot simply count the amount of transition metal in our catalyst as a large proportion has been shown to be in inactive phases or buried within the structure^10^. Although cyanide, thiocyanide or hydrogen sulfide do interact or poison Fe–N/C catalysts, these species could not be successfully utilized as electrochemical probes^18^,^19^,^20^.

In contrast, we find that Fe–N/C catalysts strongly interact with the nitrite anion and form a stable poisoned catalyst adduct, while a metal-free catalyst is unaffected. The stability of this adduct is remarkable and enables its use as an active site probe–once formed the adduct is not removed even after storing the electrode at OCP in electrolyte for 24 h, or by performing an ORR scan across the potential region typically used. Such stability and ability to operate during the ORR is not commonly seen for such ‘reversible' poisons, for example, CO adsorption on platinum. Moreover, under the right conditions, the catalytic activity can be completely recovered through reductive nitrite stripping at very low potentials. The stripping charge compared with the extent of the poisoning allows quantification of the number of active sites.

## Results

### Synthesis and physical characterization of the Fe–N/C catalyst

The Fe–N/C catalyst is prepared as per literature procedure with reasonable fuel cell results^14^. To ascertain whether the likely active centres in this material are atomic sites or particulate phases, high resolution transmission electron microscopy (TEM) and scanning transmission electron microscopy (STEM) images from various areas within the catalyst were recorded.

**Figure 1:**
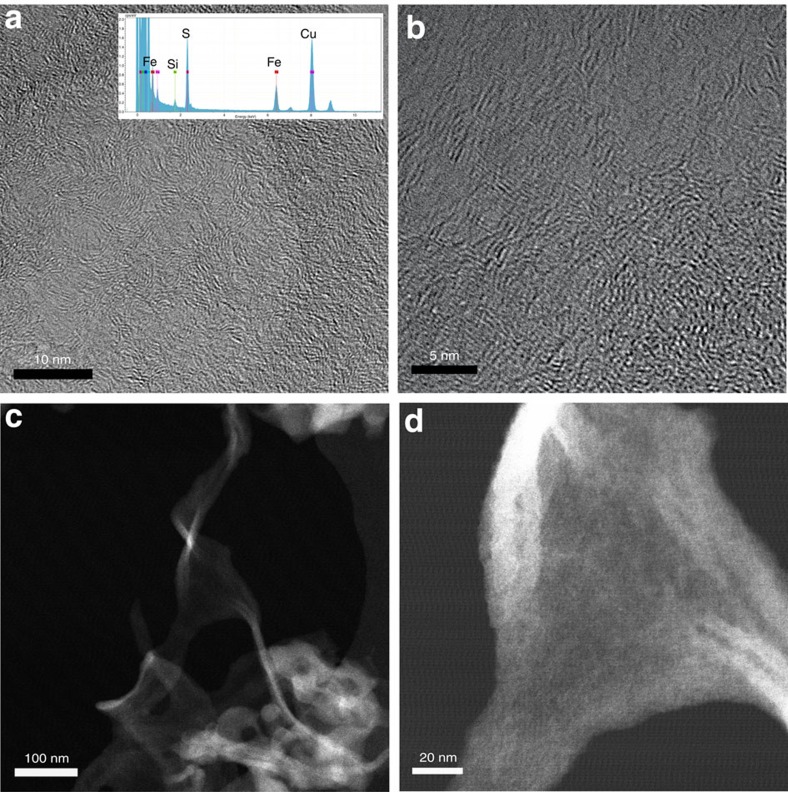
Representative TEM images showing absence of solid metal particles in Fe–N/C. (**a**,**b**) Representative high resolution TEM images of the Fe–N/C catalyst, showing the absence of solid inclusions or nanoparticles and the amorphous structure. Inset (**a**) high resolution EDS of region corresponding to image (**b**) clearly showing the presence of iron. (**c**,**d**) High resolution STEM images of Fe–N/C catalyst. See Supplementary Figs 1 to 4 for more images and discussion.

Figure 1 shows representative TEM (a and b) and STEM (c and d) images. It can be seen that there are no significant encapsulated carbide phases visible, while iron is clearly present as confirmed by high resolution energy-dispersive X-ray spectroscopy (EDS) (inset Fig. 1a). This indicates highly dispersed iron. If present in large quantities, nanoparticles should be visible in the images at this resolution, as observed for some types of Fe–N/C catalysts^5^,^21^,^22^,^23^,^24^. We therefore infer that these phases are not the predominant source of the activity and our iron sites are likely to be similar to those reported for the catalysts of the LANL and Dodelet groups^3^,^4^,^5^, namely atomic Fe–N_x_ sites, since different preparation methods lead to common sites^8^,^11^,^15^. This is also supported by the fact that no crystallinity indicative of such nanophases is detected in various different TEM images from different regions within the same material (Supplementary Figs 1–4 for more images and details)^23^,^24^. Total reflection x-ray fluorescence determines the iron content to be 1.5±0.2 wt% (Supplementary Fig. 5 and Supplementary Method 1). The Brunauer-Emmett-Teller (BET) surface area of the material was determined to be 531±1.5 m^2^ g^−1^ with an external surface area of ∼110 m^2^ g^−1^ (Supplementary Fig. 6 and Supplementary Table 1).

### ORR on the Fe–N/C catalyst across the pH scale

**Figure 2:**
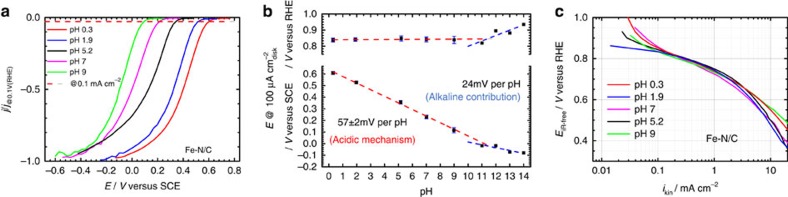
ORR on the Fe–N/C catalyst across the pH scale. (**a**) Rotating disk electrode measurements of Fe–N/C catalyst at different pH values in O_2_ saturated 0.5 M electrolytes, 1,600 r.p.m., 5 mV s^−1^; loading 270 μg cm^−2^ data corrected for solution resistance, capacitive background and different oxygen solubility and diffusivity. (**b**) Plot of the potential at a current density of 0.1 mA cm^−2^ (iR-free) versus pH (bottom of panel) versus saturated calomel electrode; linear fit shows a slope of 57±2 mV/pH in the pH range 0–9 (top of panel) corrected to RHE scale. All values become the same within the error margin in the pH range 0–9 corrected to RHE scale. (**c**) Tafel plot of mass-transport corrected currents from (**a**) corrected to the RHE potential scale. All plots collapse into one, especially at high potentials.

While nitrite poisons the catalytic activity in acid electrolyte at a pH of 0.3 as it does at higher pH electrolytes (Supplementary Figs 7–13 and Supplementary Note 1), it was necessary to resort to higher pH values for the stripping experiments to improve quantification of the stripping peak and to remove interference caused by the formation of NO and NO_2_ by acid decomposition of nitrite (Supplementary Fig. 13 and Supplementary Note 1). To validate whether the underlying catalytic mechanism is liable to be the same at higher pH values compared with the technologically important acidic activity, the ORR was studied at various pH values, which previously, to our knowledge, has only been investigated for low (0–2) and high (12–14) pH values^25^. Figure 2a shows the, iR-free, background corrected rotating disk electrode linear sweep voltammograms of the catalyst, which were normalized to the different oxygen solubility in the respective electrolytes. By plotting the current at 0.1 mA cm^−2^ versus pH, a linear plot with a slope of close to 59 mV pH^−1^ is obtained (Fig. 2b). This indicates a proton coupled electron transfer as rate determining step for the reduction of oxygen to water at high potentials. This is intriguing and might inspire approaches on how to accelerate the rate determining step. It can be seen that in the pH range between 0 and 9 the activity at high potentials (Fig. 2b top) is the same. It is known that the ORR is more facile in alkaline solution and a changeover in mechanism is likely at high pH (ref. 9). To exclude that this changeover is already occurring at pH 5.2, we extended our analysis of the activity to pH14 and found that a significant deviation from 59 mV/pH and the associated changeover in mechanism is only present above pH9 (Fig. 2b). It can clearly be seen that the pH dependence changes only at this high pH (>9) and only then the rate enhancing effect of using alkaline conditions becomes measurable. This is also evident from the increase in activity above pH 10, when compared on the reversible hydrogen electrode (RHE) scale (Fig. 2b top). Furthermore, the apparent Tafel slopes are the same within our error margin throughout the pH range (Supplementary Fig. 14 and Supplementary Table 2). Figure 2c shows that the Tafel plots of the measurement in Fig. 2a, corrected to the RHE scale, and corrected to kinetic currents all collapse onto one curve. This strongly suggests that the underlying catalytic mechanism is likely to be the same in this pH range and information on TOF and active site density determined at pH 5.2 can be transferred to acidic conditions. The adaptability of pH 5.2 is further supported by the same stripping pattern being seen at pH 0.3. (Supplementary Fig. 13 and Supplementary Note 1). In acid, adsorption and subsequent stripping of nitrite is complicated due to the disproportionation of the nitrous acid formed in acidic conditions (pK_a_=3.4) into NO and NO_2_. These species act as interferents and make quantification less accurate (Supplementary Fig. 13 and Supplementary Note 1), this also means that care must be taken in selecting the appropriate concentration of nitrite (Supplementary Note 1)


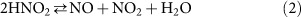


### Determining the number of active sites

**Figure 3:**
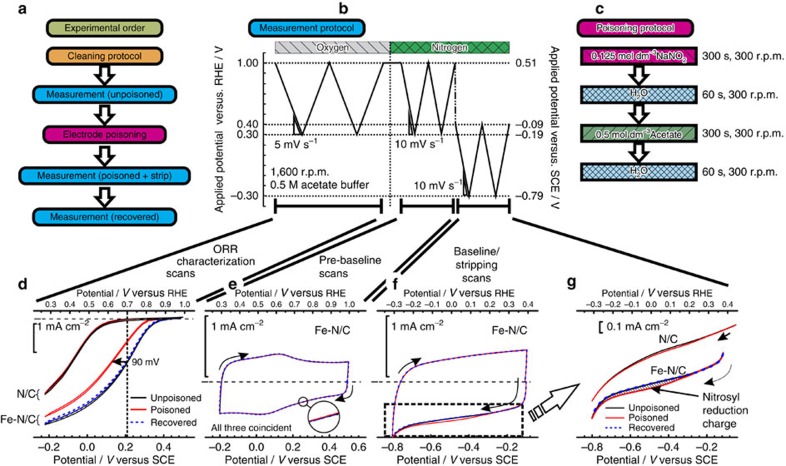
Protocol to determine catalyst site density through reversible nitrite poisoning. (**a**) Flow diagram showing steps required to assess the performance of a catalyst and determine the catalyst site density; (**b**) measurement protocol used to measure the electrochemical performance of the ORR and assess the charge associated with reductive stripping of the adsorbed nitrite; (**c**) protocol used to poison the electrode using a nitrite containing solution; (**d**) ORR performance of catalyst layer before, during and after nitrite adsorption; (**e**) wide range baseline scan (avoiding nitrite reduction area) for the catalyst layer before, during and after nitrite adsorption; (**f**) narrow baseline scan in the nitrite reductive stripping region before, during and after nitrite adsorption; (**g**) expansion of the region associated with nitrite stripping. All experiments were performed in a 0.5 M acetate buffer at pH 5.2 for Fe–N/C catalyst using a rotating disk electrode setup; loading 0.27 mg cm^−2^.

**Figure 4:**
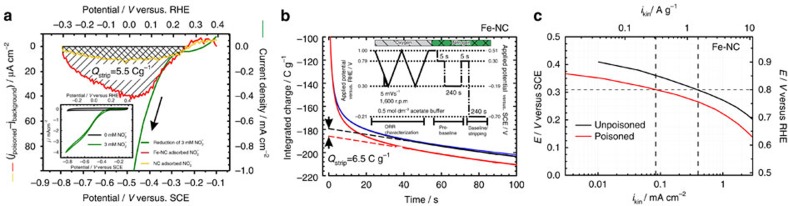
Interaction of nitrite on Fe–N/C and N/C catalysts. (**a**) Comparison between homogeneous reduction of aqueous nitrite (3 mM NaNO_2_ in acetate buffer), and excess current associated with reductive stripping of intermediate on Fe–N/C or N/C catalyst. The reductive stripping curve is produced by subtracting the unpoisoned from poisoned curve in Fig. 3g. Inset is the complete nitrite reduction curve for homogeneous nitrite in solution. (**b**) Chronoamperometric transients for determination of the reductive stripping charge for the Fe–N/C catalyst; (**c**) kinetic current density of Fe–N/C catalyst before and after the poisoning step. O_2_-saturated electrolyte, 5 mV s^−1^ background and iR-corrected rotating disk electrode experiments at 1,600 r.p.m., electrolyte: 0.5 M acetate buffer, loading: 0.27 mg cm^−2^.

To determine the number of active sites, it is necessary to perform a series of experiments (Supplementary Method 2) with the catalyst deposited on a rotating disk electrode in a 3-electrode set-up^26^. As previously described, a 0.5 M acetate buffer at a pH of 5.2 was used as electrolyte to improve reproducibility, which indicates that the nitrite reduction is sufficiently facile under this pH while the nitrite anion is sufficiently stable (Supplementary Note 1). This enables the correlation of the stripping onset potential to the nitrite reduction onset potential (Fig. 4a) and also shows that the metal-free catalyst does not appreciably reduce solution phase nitrite—implicating an iron species in the active site (Supplementary Figs 15–17). A saturated calomel electrode was used as reference electrode and the potentials were converted to the RHE scale (Supplementary Fig. 18). Figure 3a depicts the order of experimental steps. After the electrode has undergone a cleaning protocol, a background scan is performed utilizing the steps shown in Fig. 3b. These steps not only measure the performance of the catalyst towards the ORR, but also measure the voltammetry in an oxygen free environment over both, a wide potential range, avoiding the nitrite reduction area, and a narrow potential range, more reductive region within which nitrite reduction occurs. After these preliminary scans, the electrode is poisoned following the protocol shown in Fig. 3c utilizing a nitrite concentration of 0.125 mol dm^−3^ (Supplementary Figs 19–21 and Supplementary Note 2 for further details). The protocol shown in Fig. 3b is then repeated to measure the performance of the catalyst in its poisoned state. The last sets of scans performed in the narrow, more reductive region lead to the reductive desorption of the nitrite. Finally the protocol shown in Fig. 3b is again repeated to see if the electrode has been recovered and returned to its initial state. Figure 3d–g collect the respective voltammograms from each of the respective phases of the measurements. Figure 3d shows the ORR performance of the catalyst as a function of poisoning. The catalyst performance is significantly reduced by the presence of an adsorbed nitrite intermediate, leading to a 90 mV shift in performance at 0.7 V (RHE). Thus, activity of the catalyst is reduced to <20% of its unpoisoned state. Following the stripping process, the ORR performance is totally recovered. Figure 3e shows that there is no discernible difference to the electrode voltammetry over a wide potential range which avoids the nitrite reductive stripping region—all sweeps overlap each other. In contrast, when the potential is swept to a lower potential, Fig. 3f, there is an excess in cathodic charge only when the electrode has been pre-exposed to nitrite. Furthermore, this excess charge disappears on subsequent scans, and the scans are perfectly coincident with those taken before exposure to nitrite. This region is shown in greater detail in Fig. 3g, and the excess charge is perfectly correlated with the electrode being pre-exposed to nitrite during the poisoning protocol. As the catalytic activity is completely recovered after the stripping CV is performed (Fig. 3d), the amount of stripped charge is directly correlated to the decrease and recovery of the catalyst performance. Moreover, the same experiments performed on a metal-free N/C catalyst (∼60 p.p.m. Fe) do not show a significant poison effect or a significant stripping charge (Supplementary Fig. 22), nor homogeneous nitrite reduction (Supplementary Fig. 17). This indicates that nitrite interacts with the metal centre of the Fe–N/C catalyst. Therefore the determination of the intrinsic catalytic activity induced by the metal is possible.

Figure 4a shows the difference in current between the stripping peak and background (that is, difference in curves shown in 3(g) for both the Fe–N/C and N/C catalyst (repeat experiments provided in Supplementary Fig. 23). It can be seen that there is some stripping charge for the N/C catalyst, and we assign this to residual iron in the sample (Supplementary Figs 24 and 25, Supplementary Tables 3 and 4 and Supplementary Note 3 for further analysis). Also plotted is the homogeneous reduction of nitrite on the same catalyst when nitrite is present in the acetate buffer at a concentration of 3 mM. It can be seen that there is a clear coincidence between the stripping peak and the beginning of nitrite reduction, strongly suggesting that they are the same process, Fig. 4a main figure and inset. The stripping charge can alternatively be determined by stripping chronocoulometry, Fig. 4b (Supplementary Method 2 for further details and Supplementary Fig. 26 and Supplementary Table 5 for repeats). A comparison of results for the repeats is in Supplementary Table 5. For metal-free materials, the information is provided in Supplementary Figs 24 and 25, in Supplementary Tables 3 and 4 and in Supplementary Note 3). After preparing the electrode in the same manner as described in Fig. 3, the electrode potential is stepped from 0.79 to −0.21 V versus RHE. Similarity of our active site to the behaviour of iron haem complexes towards nitric oxide and nitrite is assumed. Hence, it is assumed that the nitrite ligand is transformed to a nitrosyl ligand upon cycling with a net reaction of 27,28:


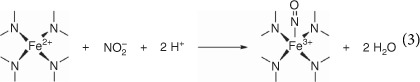


It is further assumed that the stripping product is ammonia and therefore a transfer of 5 electrons per stripped molecule is tentatively used here^27^,^28^.


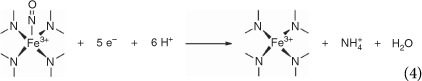


A detailed analysis of the nitrite reduction and electron transfer number is in preparation. The number of active sites is then equal to the number of stripped molecules. The areal site density (SD) is the number of active sites normalized to the surface area. We use the external surface area here, as the micropores might not be electrochemically accessible^29^.


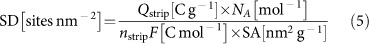


Where *Q*_strip_ is the excess coulometric charge associated with the stripping peak (Fig. 4a,b), *n*_strip_ is the number of electrons associated with the reduction of one adsorbed nitrosyl per site, and SA is the surface area of the material. Likewise, the gravimetric site density (MSD), which is the amount of active sites normalized to the mass, is calculated from:


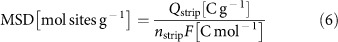


Figure 4c shows the difference of the kinetic current between the poisoned and the unpoisoned state of the Fe–N/C catalyst. By extracting the difference in kinetic current Δ*i*_k_ at 0.8 V versus RHE, the mean TOF of all different nitrite sensitive active sites with respect to electrons can be obtained via:


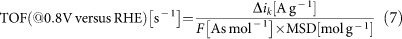


To confirm the generality of the stripping method, a bimetallic iron cobalt catalyst (Supplementary Method 3 for preparation, Supplementary Figs 27–29, Supplementary Tables 6 and 7), and a cobalt catalyst (Supplementary Figs 30 and 31 and Supplementary Note 4) have also been tested. This shows that provided the catalyst is not highly active for hydrogen evolution, which would mask the stripping charge, the method should work for the vast majority of Fe–N/C catalyst. For our Fe–N/C catalyst we arrive at an MSD of 12±2 μmol g^−1^ (7.2 × 10^18^ sites g^−1^). Interestingly, if iron is assumed as the active site, the utilization compared with the total iron content (as determined by total reflection x-ray fluorescence) is only ∼4.5%, which suggests that strategies could be implemented to increase the activity by making more iron sites accessible. The residual unutilised or undetected iron might be present as either Fe–N_x_ sites which are buried within the structure, thus inaccessible or inactive and/or buried iron or iron based nanoparticulate phases (although we have not detected such phases in our system). It is furthermore possible that the residual ∼20% activity of the poisoned catalyst is caused by a second type of iron containing active site which is less sensitive to nitrite (see Supplementary Fig. 32 for reduction of performance to almost background level). These can either be in the form of nanophases or specific types of Fe–N_x_ sites with a higher affinity to oxygen as compared with nitrite (Supplementary Note 2). The steric effects on the relative affinity of different substrates on iron centres is documented for enzymes^30^,^31^. In the presence of nitrite in solution, the catalytic activity is almost completely decreased to metal-free level suggesting that nitrite also interacts with these other active sites, be it not as strong (Supplementary Fig. 32). Interestingly, Lefevre *et al*.^32^,^33^ found that the abundance of less active ORR sites amounts to 20%, where the remaining 80% was assigned to a more active (and more desirable) ORR site. This coincides with the 80% decrease in ORR activity at 0.8 V versus RHE observed in the present work upon poisoning the Fe catalyst with nitrite. Follow-up studies using *ex situ* techniques in combination with the nitrite probe might identify which site-type is more active and hence more desirable to pursue. It has been shown that this type of material can contain up to five different types of Fe–N_x_ sites and different TOFs have been assigned to those^8^,^11^,^32^. Due to the chemical similarity, it is likely that several of these different sites interact with nitrite. Therefore, this method will yield an average TOF for all different sites combined. Nevertheless, it is now possible to track whether a higher mean TOF is possible by enriching the catalyst with a certain type of site, as suggested by Mössbauer measurements^11^. Our site density is 0.07±0.01 nm^−2^ (0.02±0.002 nm^−2^ with respect to the total surface area). The mean TOF at 0.8 V versus RHE is 1.6±0.2 s^−1^, which is in excellent agreement with the TOF for these types of active sites as reported by Sahraie *et al*.^10^ which was determined to be ∼1.5 s^−1^ by a combined Mössbauer/chemisorption study and supports the validity of our method (further details on statistics of the measurements are provided in Supplementary Table 5).

To summarize, we present a methodology to obtain crucial catalyst properties, namely the mean TOF and active site density. The method is easy to conduct and requires no special equipment on top of the standard electrochemical characterization tools. Poisoned sites are stable over the long term, so the catalyst can be prepared for *ex situ* measurements, and then recovered through an electrochemical treatment. This might be very useful in pinpointing the sites of relevance to the production of the majority of current which could be probed through the use of Mössbauer and EXAFS spectroscopy. Then one could track different catalysts and analyse which ones show variations in MSD and which variations in TOF. Because the adduct formed with nitrite is stable under oxidative systems, it is possible to measure the performance of the poisoned catalyst during the ORR, something which is not possible during, for instance, the CO-dependent poisoning of platinum. This approach may allow researchers to identify hitherto inaccessible trends and could significantly speed up the improvement of Fe–N/C catalysts.

## Methods

### Catalyst synthesis

The catalyst Fe–N/C was synthesized by dissolving 1.0 g (6.4 mmol) of 1,5-diaminonaphthalene (97%, Alfa Aesar), 1.0 g (4.4 mmol) of (NH_4_)_2_S_2_O_8_ (98%, Sigma-Aldrich) and 35.6 mg of FeCl_2_·4H_2_O (99%, Sigma-Aldrich) in 250 ml of ethanol (absolute, VWR). The solution was stirred for 24 h at room temperature. The solvent was then removed with a rotary evaporator. When dry, the resulting residue was transferred to an alumina boat (11-cm long by 2-cm wide by 1-cm deep, ∼10 ml of volume capacity) and heat treated at 950 °C for 2 h, after reaching the end temperature, in a tube (quartz) furnace (Carbolite) at a heating rate of 20 °C min^−1^. This heat treatment was performed in an inert atmosphere, under a continuous flow of nitrogen (50 ccm). After cooling down under nitrogen, the resulting material was removed from the quartz boat and refluxed in 0.5 M H_2_SO_4_ for 8 h, to remove any soluble metal phases. The material was then filtered and dried. The dried powder was then subjected to a second heat treatment at 900 °C for 2 h after reaching the target temperature at a heating rate of 20 °C min^−1^ under nitrogen and allowed to cool as above. The resulting powder was then ready to use. The catalyst N/C was synthesized in the same way without the addition of the metal salt.

### TEM/STEM/EDS

Transmission and scanning transmission microscopy images were recorded on a FEI TITAN 80/300 equipped with a Quantax EDS system from Bruker. Samples and TEM grids were dried under vacuum before use, to minimize contamination.

### Electrochemical stripping experiments

Measurements were conducted with a Rotating Ring Disk Electrode (Pine Instruments, model AFE6R1AU, with a mirror polished glassy carbon disk and rotator model AFMSRCE), the catalyst was deposited on the glassy carbon disk as per literature procedure^26^. The ink utilized consisted of 1 wt% catalyst in a 1:1 volume ratio mixture of IPA (VWR):H_2_O (MilliQ 18.2 MΩ cm) with a Nafion to catalyst weight ratio of 1:1. This composition was found to give a uniform catalyst layer. A loading of 0.27 mg cm^−2^ was chosen as loading in all experiments, as it was found to give a good catalyst layer. Furthermore, it was a compromise between a relatively thin layer and a sufficient activity, to ensure adequate signal to noise ratio. A custom made three compartment electrochemical glass cell was used. The reference electrode was ionically connected to the main compartment of the electrochemical glass cell via a Luggin–Haber–Capillary. For measurements in 0.5 M H_2_SO_4_, a RHE (GaskatelHydroFlex) was used. For measurements in the higher pH electrolytes a saturated calomel electrode (VWR) was used and the potentials with respect to the RHE scale were determined by measuring the change from hydrogen evolution to hydrogen oxidation in the respective H_2_-saturated electrolyte on a platinized platinum wire. A glassy carbon rod was used as counter electrode and ionically connected to the main compartment of the glass cell through a porous frit. Glassy carbon was used instead of Pt to avoid contamination with catalytically active precious metals. A potentiostat (Autolab, PGSTAT20) was used for potential or current control during the electrochemical measurements. Ultrapure gases, nitrogen, oxygen and hydrogen (BIP plus-X47S, Air products) were used. Electrolytes were prepared in ultrapure water (MilliQ 18.2 MΩ cm). 0.5 M H_2_SO_4_ from 95% sulfuric acid (Aristar, VWR), 0.5 M phosphate buffer pH 2 from phosphoric acid (AnalR Normapur, VWR) and NaH_2_PO_4_ (AnalR Normapur, VWR), 0.5 M acetate buffer pH 5.2 from sodium acetate (99%, Sigma-Aldrich) and glacial acetic acid (AnalR Normapur, VWR), 0.5 M phosphate buffer pH7 from NaH_2_PO_4_ (AnalR Normapur, VWR) and Na_2_HPO_4_ (AnalR Normapur, VWR) and 0.5 M borate buffer from boric acid (ACS reagent, 99.5%, Sigma-Aldrich) and NaOH (AnalR Normapur, VWR). The pH was adjusted with 0.5 M NaOH and confirmed with a ROSS Ultra Glass pH Electrode (Orion 8102BNUWP). The normalized current density was corrected to account for the different solubility and diffusivity of oxygen in the different electrolytes. We used the current at 0.1 V to determine a ‘normalized' current to which all the currents were ratioed. Therefore the normalized current density was obtained by: 
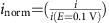
, where *i* is the geometric current density and *i*_norm_ the normalized current density. The kinetic current density was estimated as follows; 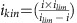, where *i*_lim_ was taken as the current density achieved @0.1 V versus RHE. The error introduced due to the deviation from the theoretical limiting current in the respective electrolyte, which was caused by insufficient activity of the catalyst at this loading, was found to be small (<1.5%) at the potential of interest (0.8 V versus RHE). It was found necessary that a cleaning protocol was performed before the stripping experiments to achieve a stable baseline, full details are provided in Supplementary Methods 2. The procedure consisted of extensive cycling, alternating between N_2_-saturated electrolyte at 100 mV s^−1^ (20 cycles) and 10 mV s^−1^ (10 cycles) and O_2_-saturated electrolyte at 5 mV s^−1^ (6 cycles), in the potential window 1.05 to −0.4 V versus RHE. This was repeated until stable non changing oxygen reduction performance and cyclic voltammograms under nitrogen were achieved (3–4 times). Where iR-free potentials (*E*_iR-free_) are reported the potential (*E*) was corrected to be *E*_iR-free_=*E*−*I* × *R*, where *I* is the measured current and *R* the solution and lead resistance, as determined by electrochemical impedance spectroscopy (FRA module, Autolab, PGSTAT20) as described in literature^34^.

### Data availability

The data used in the preparation of the figures in this paper are available for download at DOI:10.5281/zenodo.159501.

## Additional information

**How to cite this article:** Malko, D. *et al*. *In situ* electrochemical quantification of active sites in Fe–N/C non-precious metal catalysts. *Nat. Commun.* **7,** 13285 doi: 10.1038/ncomms13285 (2016).

**Publisher's note:** Springer Nature remains neutral with regard to jurisdictional claims in published maps and institutional affiliations.

## Supplementary Material

Supplementary Information Supplementary Figures 1-32, Supplementary Tables 1-7, Supplementary Notes 1-4, Supplementary Methods 1-3 and Supplementary References
