# Supplementary material for: In situ electrochemical quantification of active sites in Fe–N/C non-precious metal catalysts
Source: Nat Commun. 2016 Oct 31;7:13285. doi: 10.1038/ncomms13285 (PMC5095514; doi:10.1038/ncomms13285)
Supplement: Supplementary Information — Supplementary Figures 1-32, Supplementary Tables 1-7, Supplementary Notes 1-4, Supplementary Methods 1-3 and Supplementary References [file ncomms13285-s1.pdf]

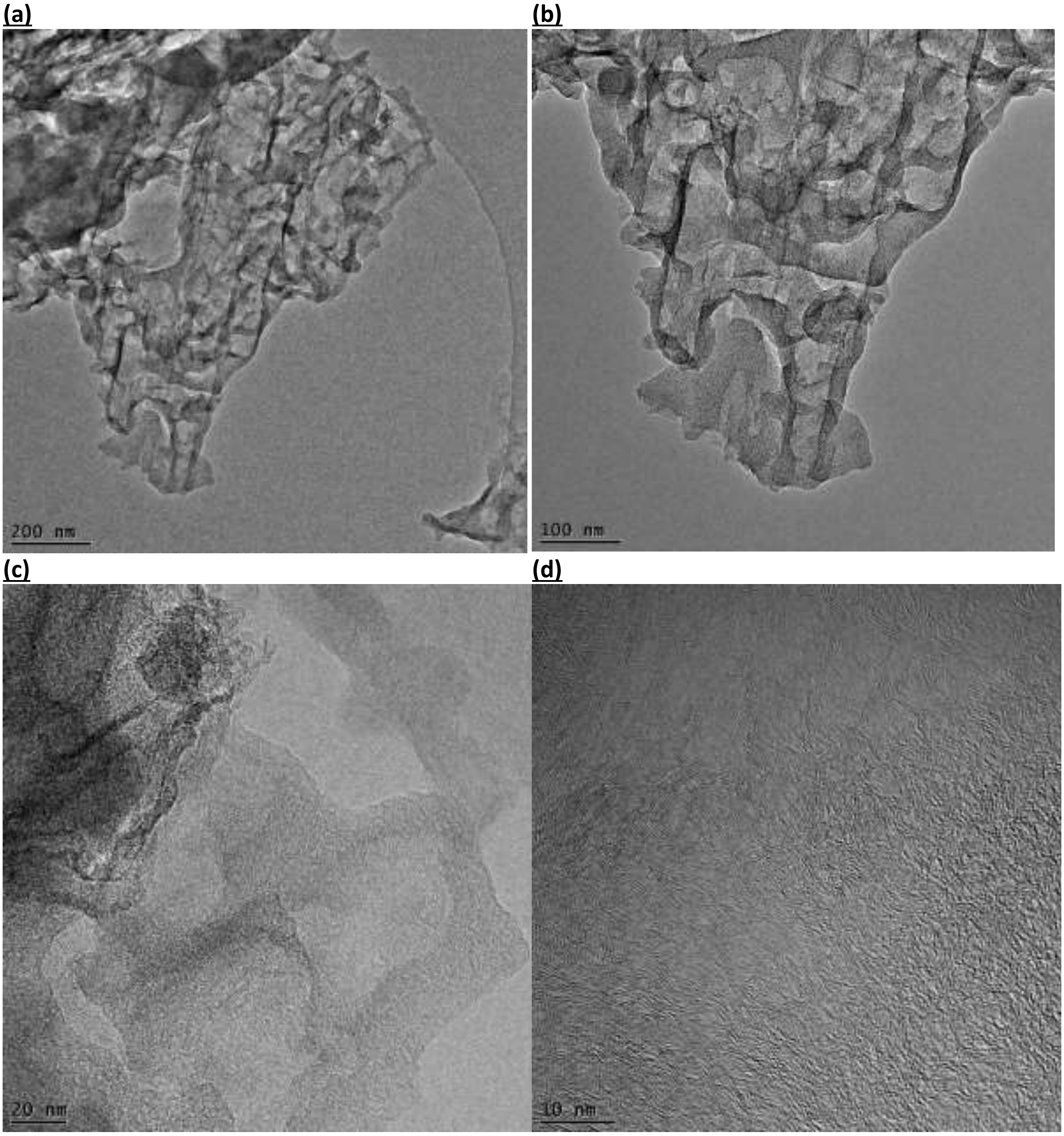

Supplementary Figure 1 ¦ TEM images of the Fe-N/C catalyst. representative TEM images of Fe-N/C catalyst at different magnifications. It can be clearly seen, while there are some slightly graphitic domains there are no particulate or encapsulated particulate phases visible.

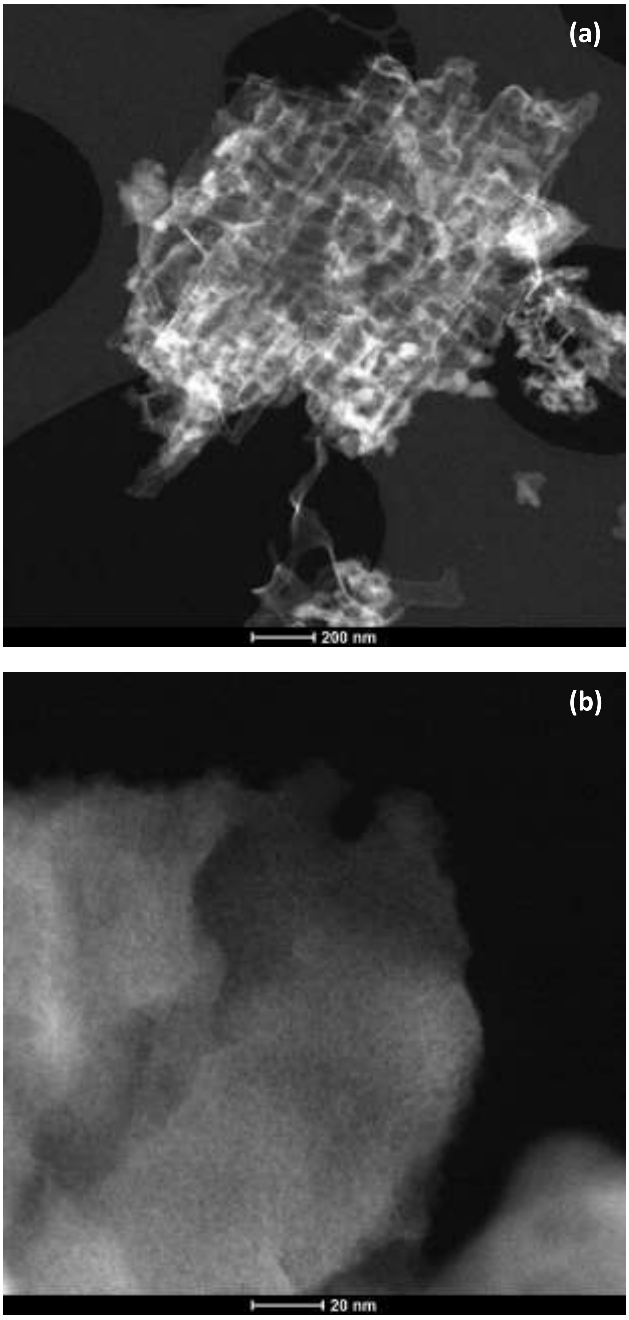

Supplementary Figure 2 ¦ STEM images of the Fe-N/C catalyst. Representative STEM images of Fe-N/C catalyst at different magnifications. Again, it can be clearly seen, while there are some slightly graphitic domains there are no particulate or encapsulated particulate phases visible.

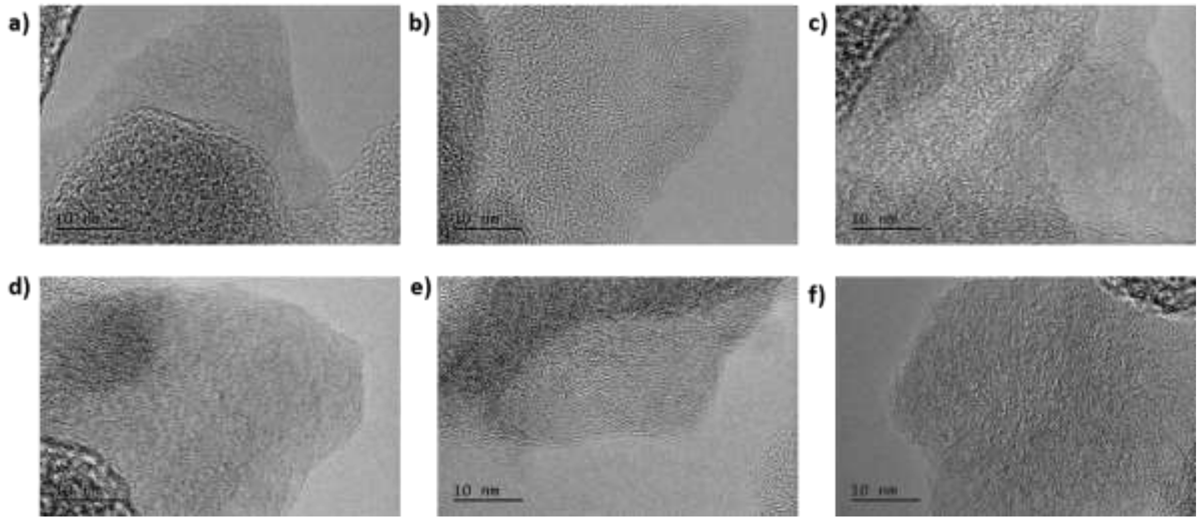

Supplementary Figure 3 ¦ HRTEM images of the Fe-N/C catalyst. a) – f) High resolution TEM images from various different regions within the same Fe-N/C material. (dark areas at the corner of a), c) d) and f) from the TEM grid.). The material seems to be highly amorphous. It can be clearly seen that no significant crystallinity is present which could indicate a significant amount of nanoparticulate phases.

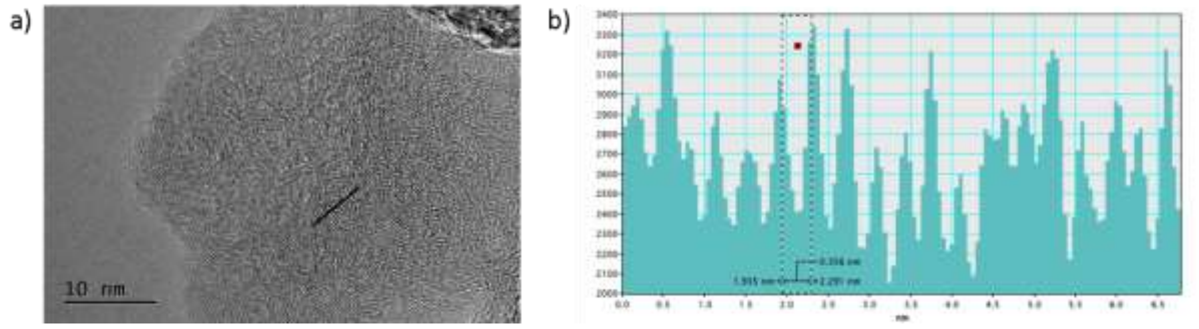

Supplementary Figure 4 ¦ TEM-based analysis of the Fe-N/C catalyst. a) TEM image of Fe-N/C material. b) Representative analysis of the lattice spacing of some ordered domains within the material. The lattice spacing of ~0.356 nm is typical of amorphous carbon with some short range order. Crystalline nanoparticulate iron, iron-carbide or iron-nitride phases would show lattice spacings below 0.3 nm. No indication of such crystallinity is found in any of the images of Supplementary Figures 1 to 3. The analysis presented here is intended to demonstrate that the Fe-N/C catalyst utilised contains most likely the same highly ORR active sites reported in literature, namely different $Fe-N_x$ species. Even though nanoparticulate phases, as reported in other materials, might be present, these do not seem to be the predominant phase. Such phases would be evident as crystalline dark areas within the TEM images and exhibit lattice spacings below 0.3 nm.[1-3] Judging from all the presented images there is no evidence of any such crystalline phases. Where shorter range order is observed (i.e. 0.356 nm), the line spacing is in agreement with the values typically found in ordered domains within less crystalline/amorphous carbon as seen above[4]

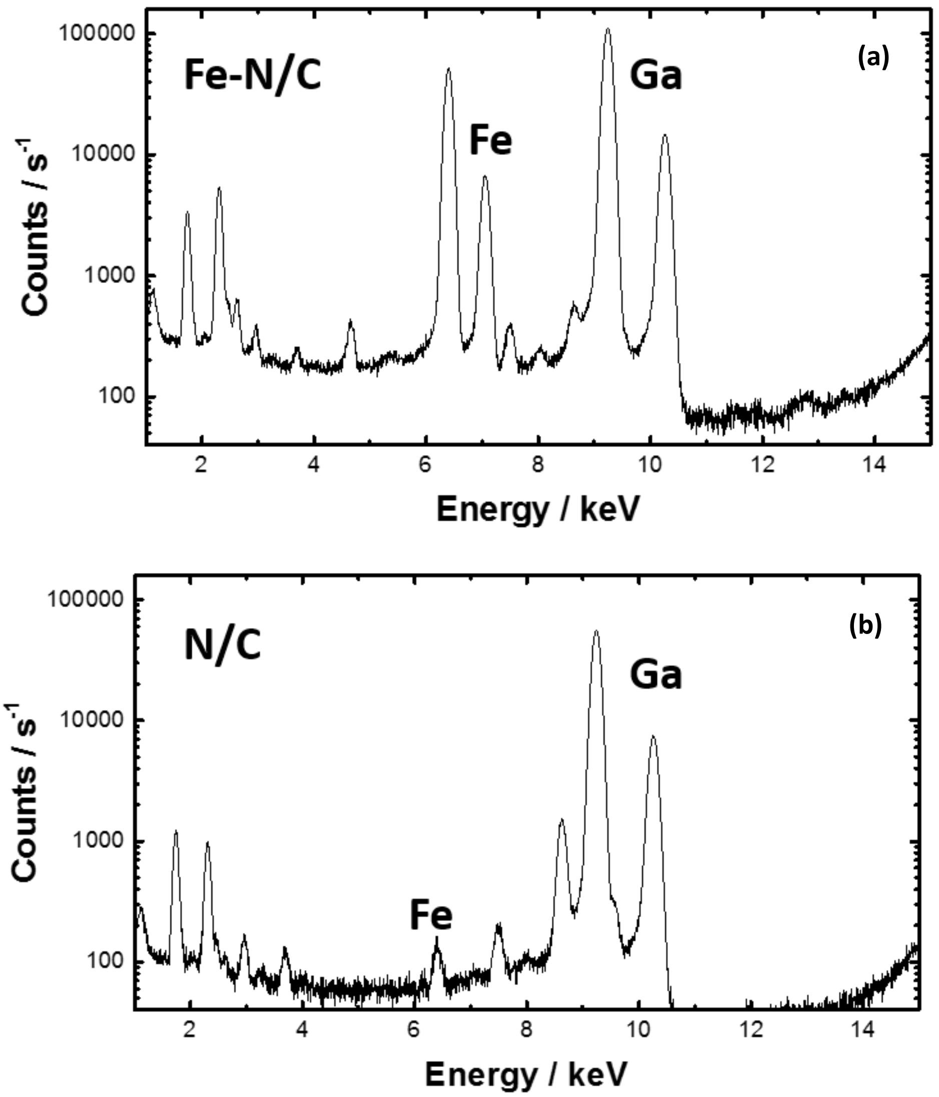

Supplementary Figure 5 ¦ TXRF spectra of Fe-N/C versus N/C catalyst in order to determine iron content and other trace metal species. (a) Total reflection x-ray fluorescence (TXRF) spectrum of Fe-N/C catalyst. (b) TXRF spectrum of N/C catalyst. See **Supplementary Method 1** for TXRF experimental details.

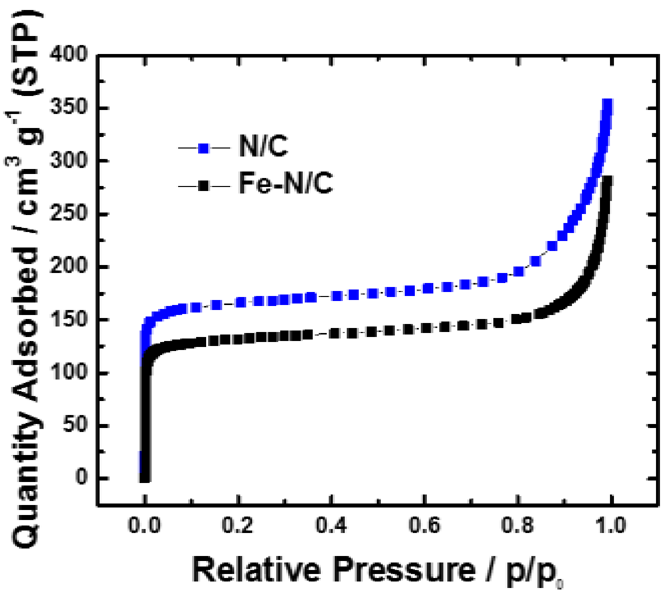

Supplementary Figure 6 ¦ Nitrogen adsorption isotherm of Fe-N/C and N/C catalysts. Nitrogen adsorption isotherm, comparing the N/C catalyst to the Fe-N/C catalyst. N_2@77K. Nitrogen adsorption analysis was conducted on a Micromeritics Tristar II 3020. The analysis temperature was 77 K. Analysis results for the data is provided in Supplementary Table 1.

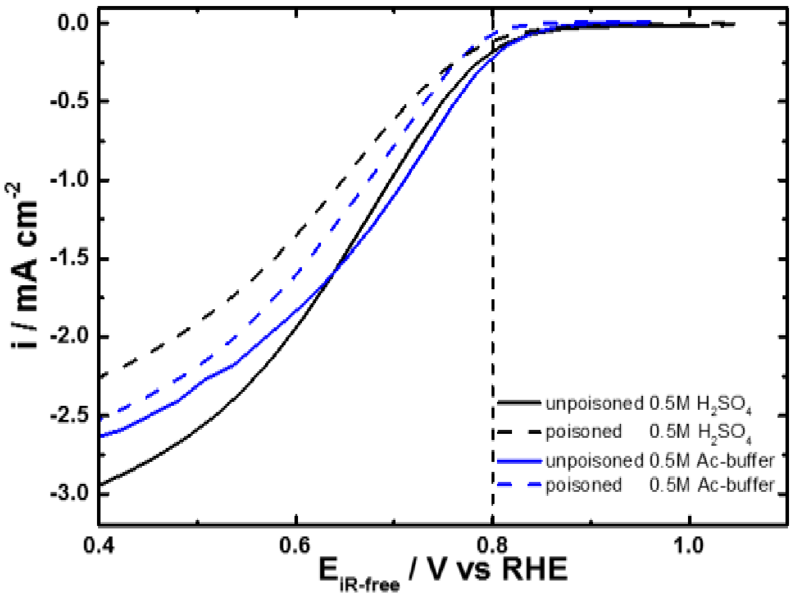

Supplementary Figure 7 ¦ ORR on nitrite poisoned and unpoisoned Fe-N/C catalyst in both H_2SO_4 and acetate buffer electrolytes with limiting current corrected current density. It can be seen that the effect of poisoning and the obtained current densities @0.8 V vs RHE are sufficiently similar to each other. This justifies the use of a higher pH electrolyte. Loading: 0.27 mg cm^{-2}, 1600 rpm. 0.5 M Acetate buffer (pH5.2) versus 0.5M H_2SO_4. 5 mV/s, background corrected, normalised to the same limiting current and iR-corrected. RDE measurements of catalysts in sulphuric acid and acetate buffer showing similar poisoning behaviour by nitrite. Note that there are different limiting current densities in the two electrolytes due to the difference in solubility and diffusion coefficient of oxygen in the two electrolytes, and (to a much lesser extent) the difference of viscosity of the two electrolytes.

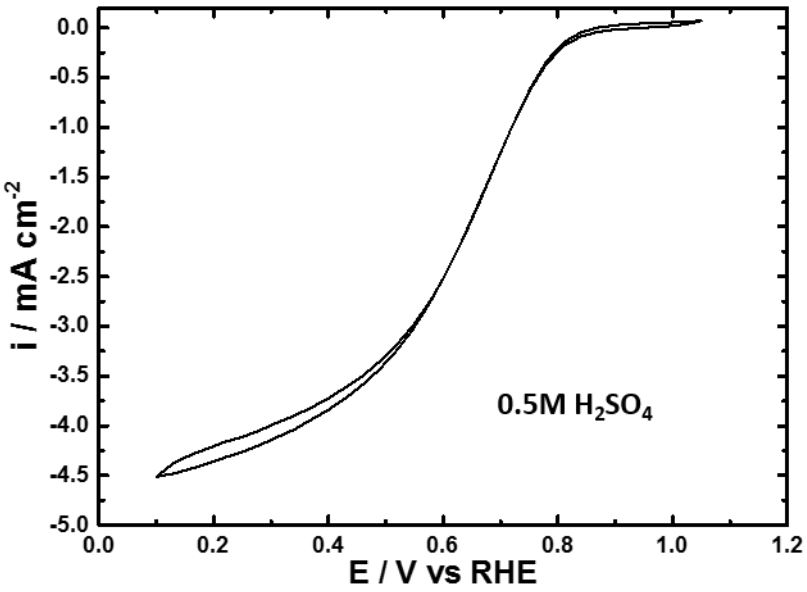

Supplementary Figure 8 ¦ Oxygen reduction reaction on the Fe-N/C catalyst in 0.5 M H_2SO_4 with uncorrected current density. Rotating disk electrode measurement in 0.5 M H_2SO_4, Loading: Fe-N/C catalyst @0.27 mg cm^{-2}, 1600 rpm, O_2-saturated. It can be seen that the current density reached in 0.5M H_2SO_4 is ~4.5 mA cm^{-2}. Although a true limiting current is not reached with this loading of 0.27 mg cm^{-2}, it was deemed more suitable to use a lower loading than usual in such system (0.6 – 0.8 mg cm^{-2}) in order to avoid inhomogeneities in the catalyst layer, which would affect the measurements.[7] A lower loading was not chosen however, as then the measured values of kinetic currents @0.8 V (RHE) and the stripping charge would become too small.

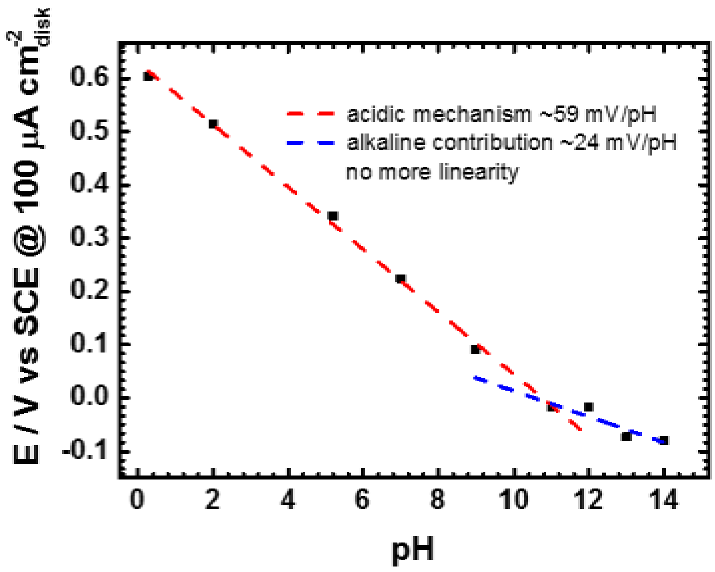

Supplementary Figure 9 ¦ Oxygen reduction reaction on the Fe-N/C catalyst across the pH scale. Plot of the potential at a current density of 0.1 mA cm^{-2} (iR-free) versus pH. (bottom) versus SCE; plot shows 2 distinct regions. The first region pH 0 - 10/11 shows a linear relationship with a slope of 59 mV/pH (red fit). Second region shows a clear deviation from the first slope and deviation from linear behaviour (blue fit). The pH dependence becomes less pronounced 24 mV/pH (blue line). O_2 saturated 0.5 M electrolytes, 1600 rpm, 5 mV/s, loading 270 µg cm^{-2}. It is clear from these data that there are 2 distinctly different pH dependencies of the ORR over the Fe-N/C catalyst. The first one shows a linear behaviour with a slope of 59 mV/pH at low pH values, while the second one shows a clear decrease of the pH dependence at higher pH. This indicates that at higher pH there is a changeover of mechanism. The decrease in pH dependence and the low pH dependence between pH 13 and 14 (only ~7 mV) indicates a significant contribution from a pH independent pathway. This is in line with suggestions of Ramaswamy et al. that an outer sphere mechanism might contribute under highly alkaline pH values, explaining the often observed high activity of catalysts towards the ORR at high pH values.[8] This however confirms as well that this changeover only occurs under highly alkaline conditions (pH>10) and the mechanism at pH 5.2 should be solely inner sphere and therefore use the same active site as the mechanism at the technologically important pH 0.3.

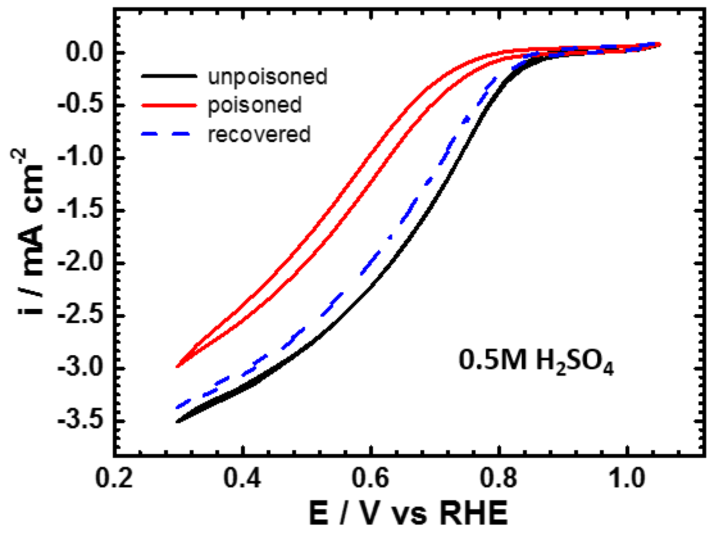

Supplementary Figure 10 ¦ Poisoning of the Fe-N/C catalyst in a 0.5 M H_2SO_4 electrolyte. Rotating disk electrode measurement of Fe-N/C catalyst before and after poisoning and recovery. Loading: 0.27 mg cm^{-2}, 1600 rpm, O_2-saturated electrolyte, 0.5 M H_2SO_4, 5 mV/s. Data in this figure show the effect of the poisoning protocol on the performance in 0.5M H_2SO_4. The same procedure as for the measurements in pH 5.2 was used, however with the difference of changing 0.5M acetate buffer to 0.5M H_2SO_4. A similar behaviour as for acetate buffer @pH5.2 is observed (Figure 3, main text). However under these condition the activity is not recovered to 100%.

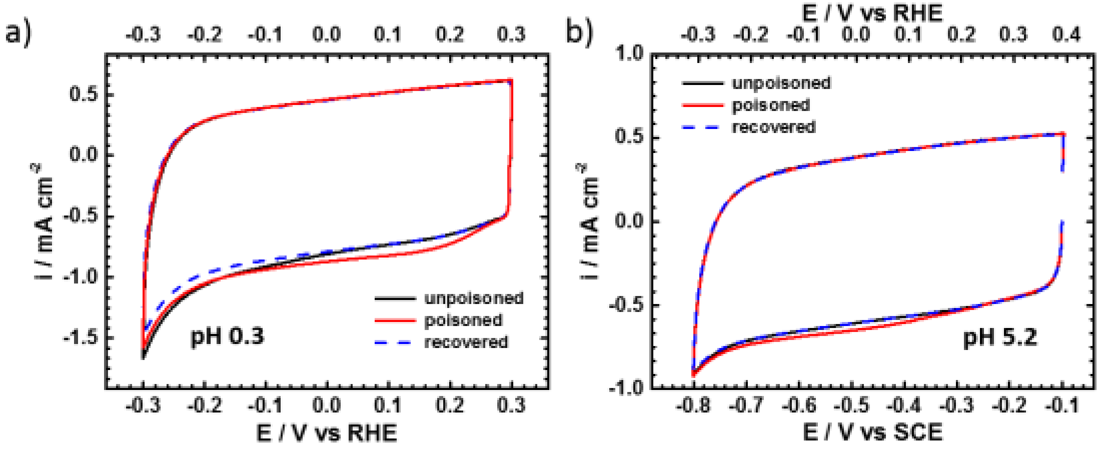

Supplementary Figure 11 ¦ Comparison of stripping experiment performed in the presence of acid compared to that performed at pH 5.2 on Fe-N/C catalyst. (a) Stripping voltammetry after ORR (in acid) performed in 0.5 M H_2SO_4; (b) Stripping voltammetry after ORR (in acid) performed in pH 5.2 buffer; Loading: 0.27 mg cm^{-2}, 1600 rpm, O_2-saturated electrolyte, 10 mV/s. Comparing the stripping pattern in 0.5M H_2SO_4 (part (a) and 0.5M Acetate buffer (part (b), it is clear that there is a mismatch between the unpoisoned and the recovered trace, indicating a change in capacitance of the system in the acidic conditions. This makes the extraction of quantitative data challenging a low pH challenging, while at pH 5.2 the traces overlap perfectly.

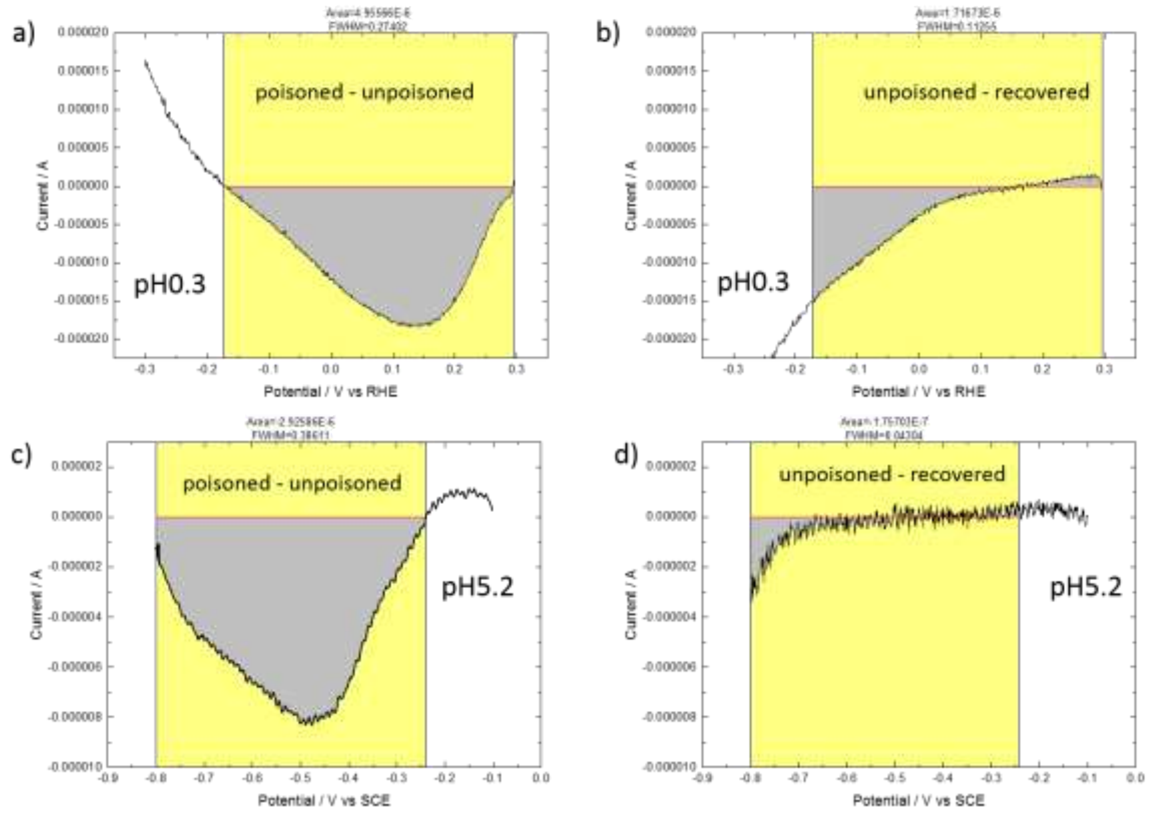

Supplementary Figure 12 ¦ Data analysis of the nitrite stripping charge of Supplementary Figure 11. Difference between capacitive traces as measured for the respective wide scan (Supplementary Figure 11) at pH 0.3 (a and b) and pH5.2 (c and d) in the stripping region. The charge difference between the poisoned and unpoisoned trace has been used to extract the stripping charge (a and c), while the difference between unpoisoned and recovered trace has been used to estimate the uncertainty due to varying capacitance. In order to estimate the uncertainty due to changes in the baseline upon poisoning, the excess charge of the poisoned to the unpoisoned trace in the region of interest ΔQ_{pu} from the scans performed in Figure 4.5 has been compared to the excess charge in the same region between unpoisoned and recovered trace ΔQ_{ur}. Comparing (b) and (d) it is evident that the discrepancy of unpoisoned and recovered trace and unpoisoned trace is significant at pH 0.3 while it is negligible at pH 5.2. The uncertainty in determining the charge due to baseline inaccuracy $\Delta Q_{ur}/\Delta Q_{pu}$ is at pH 0.3 ~35% and at pH 5.2 only ~5%.

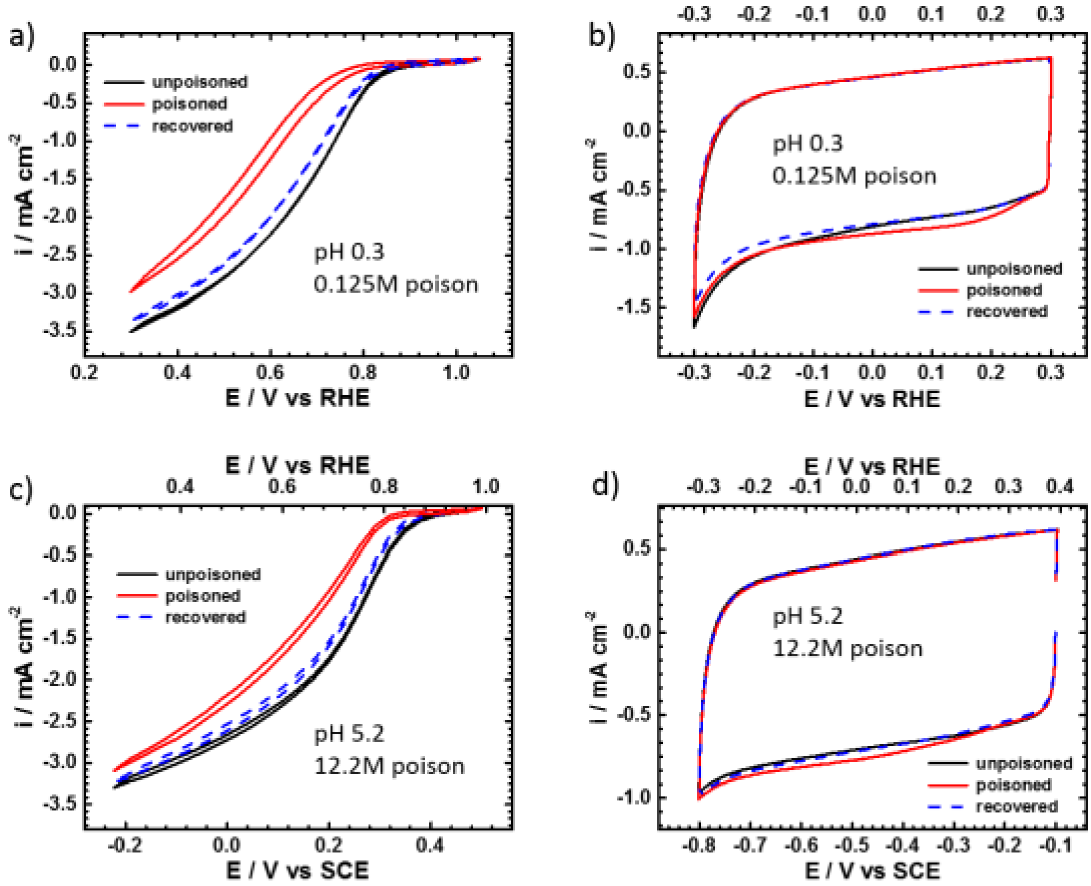

Supplementary Figure 13 ¦ Oxygen reduction reaction and nitrite stripping on the Fe-N/C catalyst in both 0.5 M H_2SO_4 and 0.5 M acetate buffer. Rotating disk electrode measurement of Fe-N/C catalyst before and after poisoning and recovery. Loading: 0.27 mg cm^{-2}, 1600 rpm (a and c) O_2-saturated electrolyte, 5 mV/s. (b and d) Stripping voltammetry, N_2-saturated electrolyte, 10 mV/s. Comparing the behaviour in 0.5M H_2SO_4 (top) to 0.5M Acetate buffer (bottom). See Supplementary Note 1 for a discussion of these results and justification of the applicability of pH 5.2.

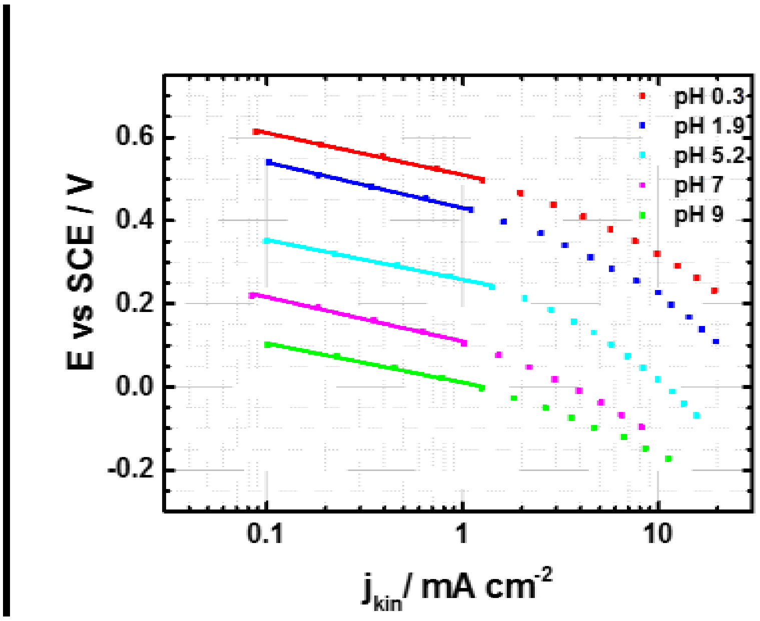

Supplementary Figure 14 ¦ Tafel-like plots of the oxygen reduction reaction on the Fe-N/C versus the SCE scale. Tafel plots corresponding to the pH dependence of ORR Including the potential at which a current density of 0.1 mA cm^{-2} is achieved and the effective Tafel slope of the catalyst in the low current region. The data are shown in Supplementary Table 2 and were averaged over three independent experiments where "independent" means that for each run a new catalyst layer from a different newly prepared ink was measured. This information was then used to produce Figure 2(b).

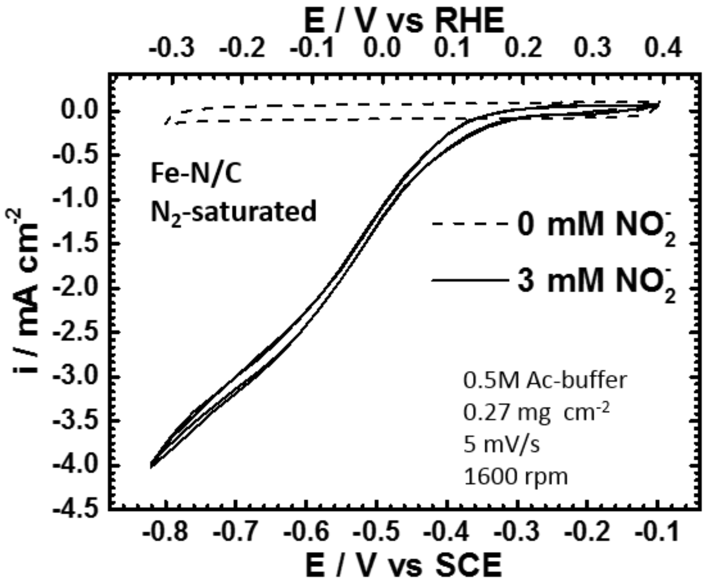

Supplementary Figure 15 ¦ Nitrite reduction reaction on the Fe-N/C catalyst in acetate buffer electrolyte showing the correlation of the nitrite reduction on Fe-N/C catalyst to stripping peak in 0.5 M Acetate buffer @ pH 5.2. Rotating disk electrode measurement: 3 mM $NaNO_2$, 0.5 M Acetate buffer (pH5.2), 1600 rpm, loading: 0.27 mg cm^{-2}, N_2-saturated, 5 mV/s.

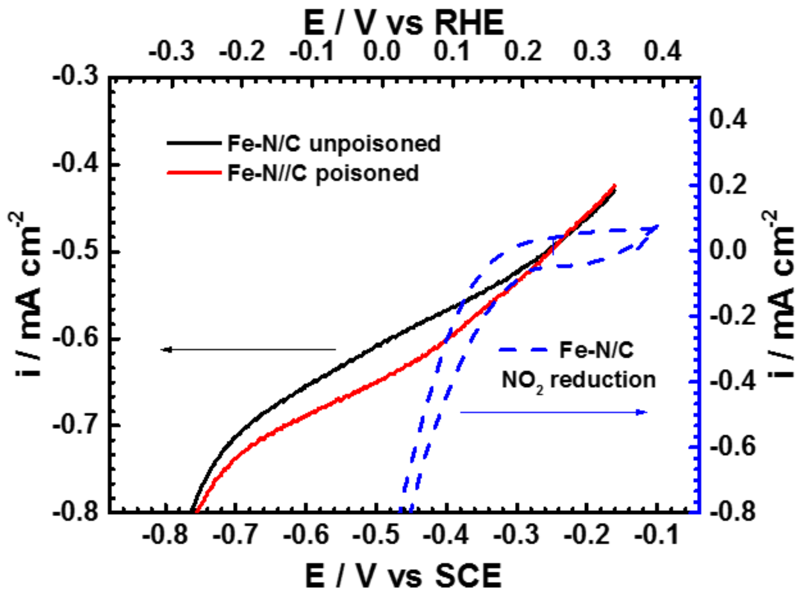

Supplementary Figure 16 ¦ Comparison of the stripping and nitrite reduction on the Fe-N/C catalyst in acetate buffer showing the correlation of the nitrite reduction on Fe-N/C catalyst to stripping peak in 0.5 M Acetate buffer @ pH 5.2. Overlay of stripping voltammetry and nitrite reduction in 0.5 M Acetate buffer (pH 5.2). It can be seen that the onset potential of stripping peak and nitrite reduction match each other. 1600 rpm, loading: 0.27 mg cm^{-2}, N_2-saturated, 5 mV/s (nitrite reduction), 10 mV/s (stripping voltammetry). The perfect coincidence of the onset in nitrite reduction with the onset of the stripping peak is a strong evidence for the same process.

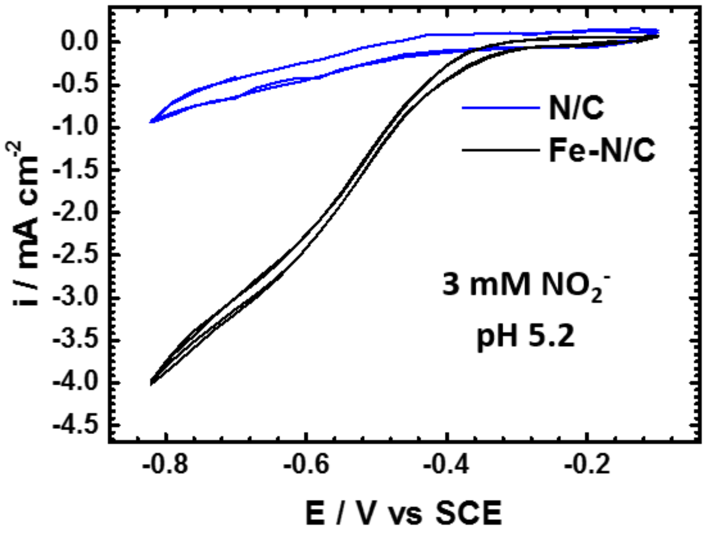

Supplementary Figure 17 ¦ Comparison of the nitrite reduction on the Fe-N/C and the N/C catalysts showing that only the metal containing catalyst is active for nitrite reduction. Comparison of the nitrite reduction over the Fe-N/C catalyst versus the N/C catalyst in 0.5 M pH 5.2 acetate buffer with 3 mM nitrite, 1600 rpm, loading: 0.27 mg cm^{-2}, N_2-saturated, 5 mV/s (nitrite reduction). It can be clearly seen that the iron containing sample is significantly more electrocatalytically active towards nitrite reduction as compared to the metal free sample.

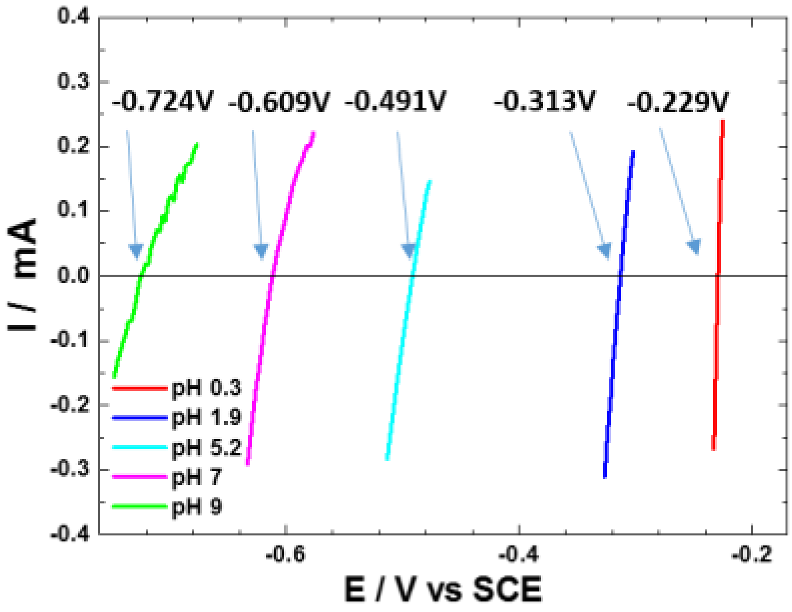

Supplementary Figure 18 ¦ Correction of the SCE scale to the RHE scale. Cyclic voltammetry of a platinized Pt wire in H_2-saturated electrolyte in order to convert SCE to RHE scale. 1 mV/s, pH0.3: 0.5 M H_2SO_4, pH2: 0.5 M PO_4-buffer, pH5.2: 0.5 M Acetate buffer, pH7: 0.5 M PO_4-buffer, pH9: 0.5 M Borate buffer.

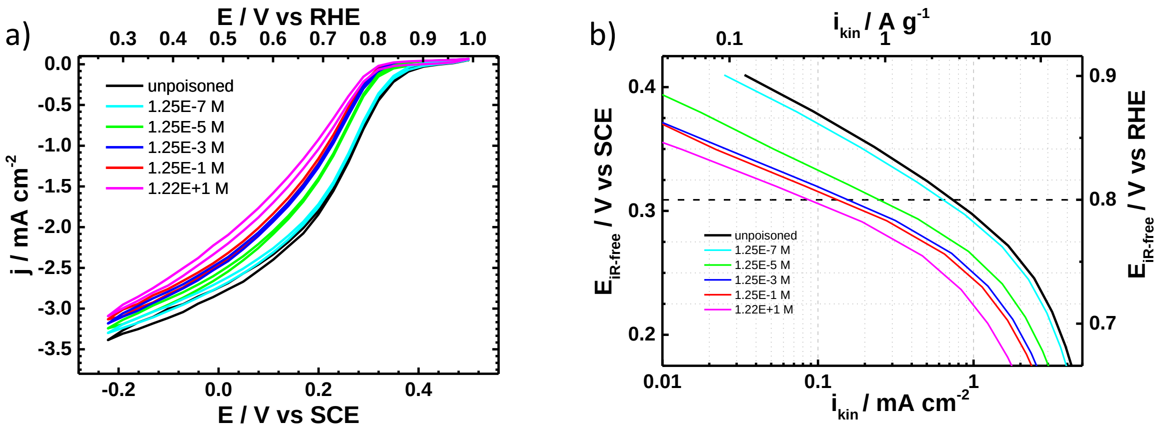

Supplementary Figure 19 ¦ Nitrite concentration effect on the ORR on the Fe-N/C catalysts in acetate buffer. a) RDE measurements of the Fe-N/C catalyst after subjecting it to the poisoning/stripping protocol at different concentrations of nitrite solution. b) Kinetic currents. Loading: 0.27 mg cm^{-2}, 1600 rpm, O_2-saturated electrolyte, 0.5 M Acetate-buffer, pH 5.2, 5 mV/s. See Supplementary Note 2 for discussion.

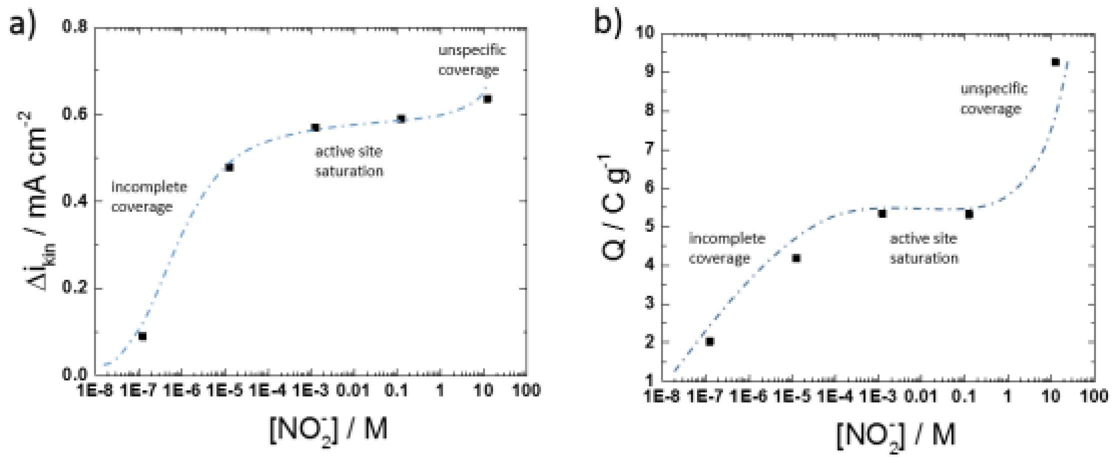

Supplementary Figure 20 ¦ Effect of the nitrite concentration on the ORR kinetic current and nitrite stripping on the Fe-N/C catalyst. a) effect of nitrite concentration on the magnitude of activity decrease b) effect of nitrite concentration on the magnitude of stripping charge. The included blue dotted lines shall aid interpretation and do not represent a mathematical fit. See Supplementary Note 2 for discussion.

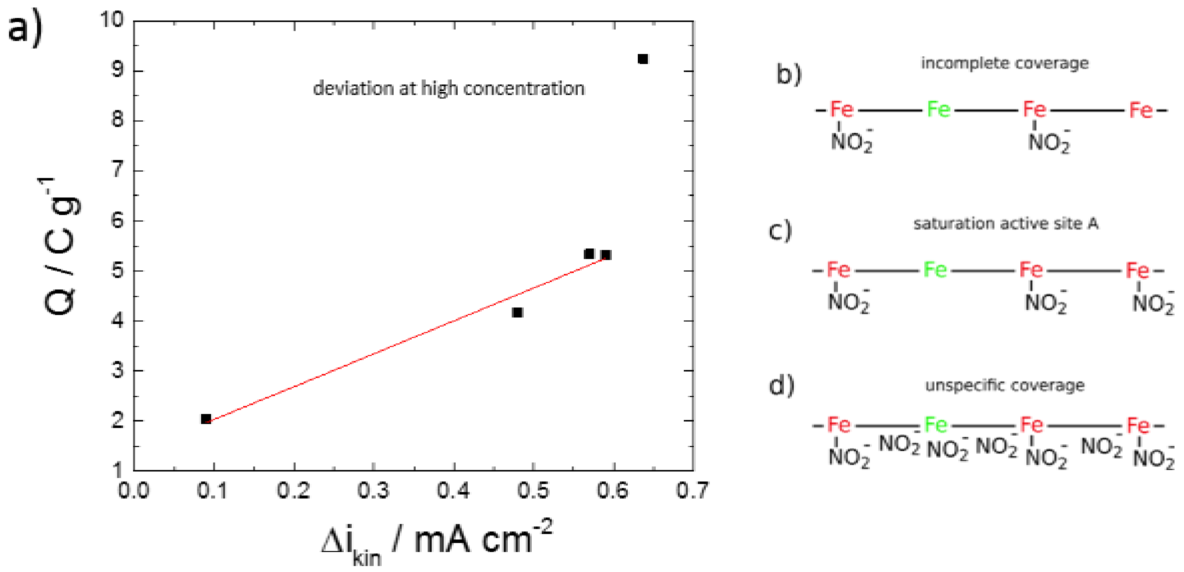

Supplementary Figure 21¦ Nitrite stripping and ORR kinetic current reduction in presence of nitrite versus nitrite coverage of the Fe-N/C catalyst active sites. a) Correlation of stripping charge to decrease in activity. b) – d) scheme to aid interpretation of the observed correlations of poison concentration, charge and activity decrease. 2 general metal based active sites are proposed. Strongly with nitrite interacting type A (red) and weakly interacting type B (green). See Supplementary Note 2 for discussion.

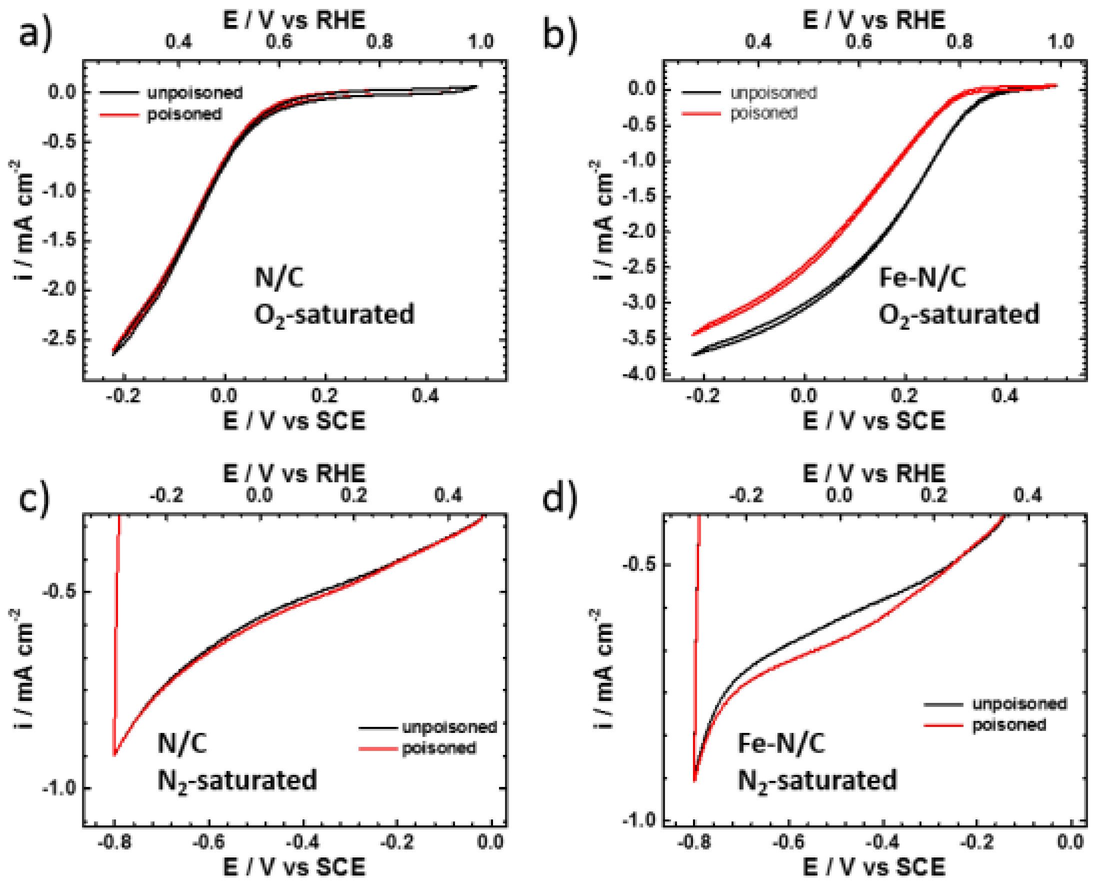

Supplementary Figure 22 ¦ Poisoning and stripping on N/C catalyst compared to Fe-N/C catalyst. Rotating disk electrode measurement of N/C catalyst a) and Fe-N/C catalyst b), 0.5 M Acetate buffer (pH5.2), 1600 rpm, loading: 0.27 mg cm^{-2}, O_2-saturated, 5 mV/s before and after subjecting to poison protocol. It can be clearly seen that the N/C catalyst is not significantly poisoned by nitrite while the Fe-N/C catalyst suffers a significant activity decrease. c) stripping voltammetry of N/C catalyst and d) Fe-N/C catalyst, before and after subjecting to posioning protocol in N_2-saturated electrolyte. It can be seen that the peak for the Fe-N/C catalyst is significantly more pronounced than for the N/C catalyst. This demosntrates that a significant amount of stripping charge is directly correlated to the metal content.

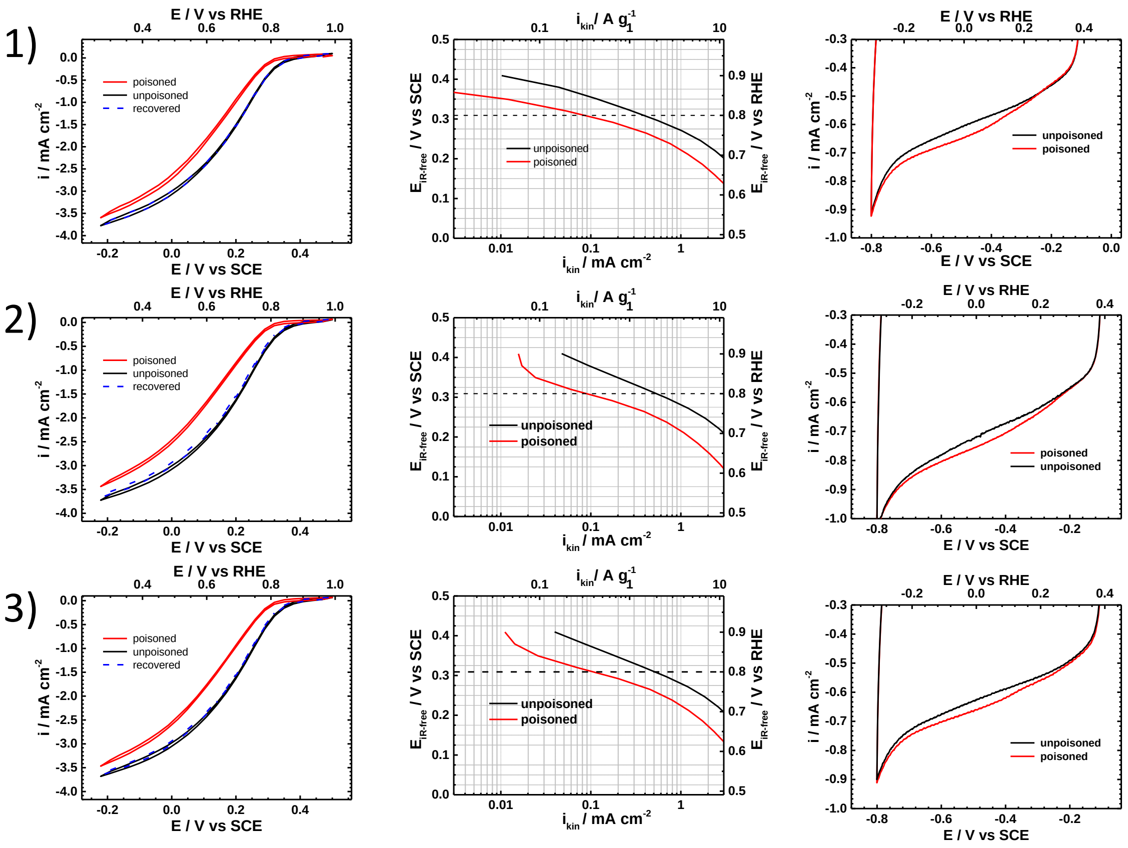

Supplementary Figure 23 ¦Repeats for stripping voltammetry of Fe-N/C catalyst at pH 5.2. 3 independent repeat measurements, demonstrating the reproducibility of the method. (left) ORR measurement in O_2-saturated electrolyte at 5 mV/s. (middle) kinetic current densities as determined from measurement left. (right) stripping voltammetry in N_2-saturated electrolyte at 10 mV/s. 1600 rpm. Loading: 0.27 mg cm^{-2}, 0.5 M acetate-buffer, pH 5.2.

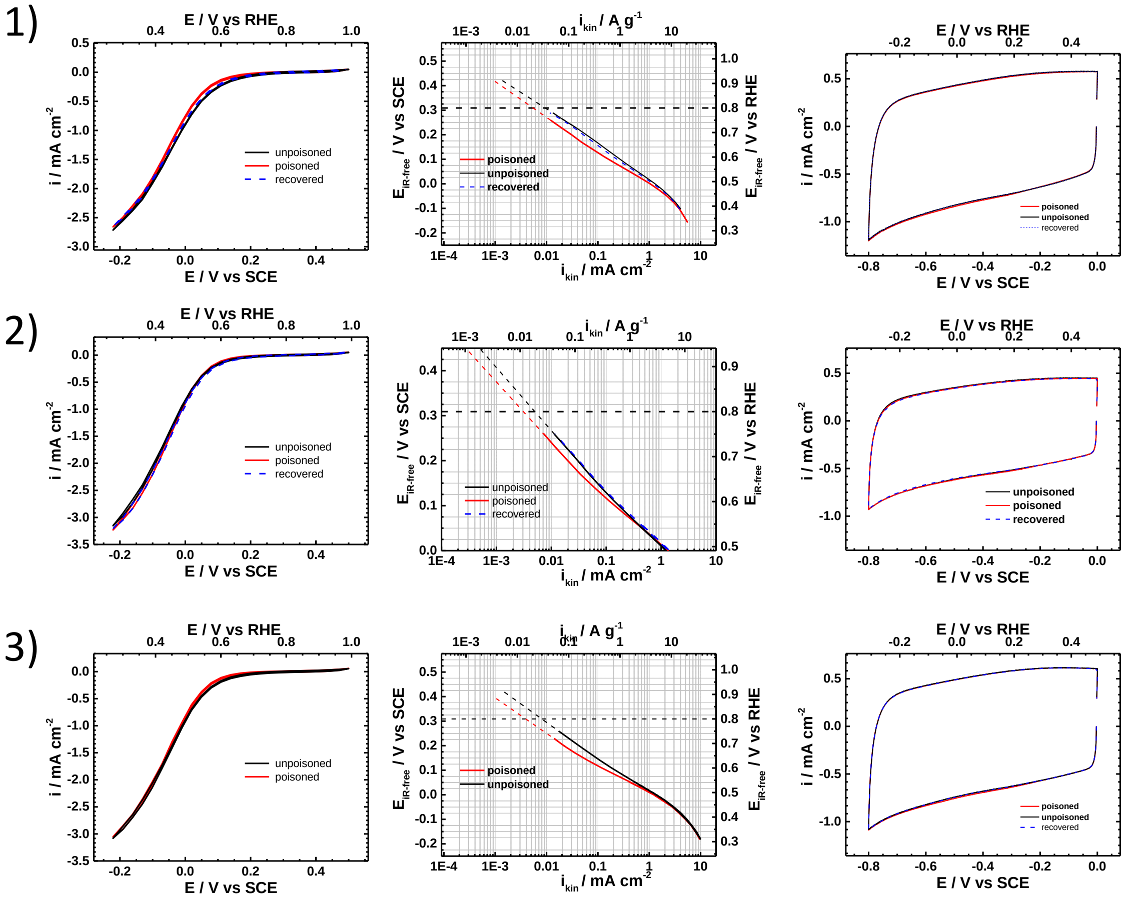

Supplementary Figure 24 ¦ Independent repeat experiments for nitrite poisoning and stripping on the N/C catalyst. 3 independent repeat measurements for stripping voltammetry on the quasi metal free catalyst, to determine the charge that is not associated with the metal centre. (left) ORR measurement in O_2-saturated electrolyte at 5 mV/s. (middle) kinetic current densities as determined from measurement left. Tafel plots extrapolated to 0.8 V vs RHE to allow comparison to the Fe-N/C catalyst. (right) stripping voltammetry in N2-saturated electrolyte at 10 mV/s. 1600 rpm. Loading: 0.27 mg cm^{-2},0.5 M Acetate-buffer, pH 5.2. See Supplementary Tables 3 and 4 for statistical analysis of data in Supplementary Figure 24 and 25. See Supplementary Note 3 for discussion on the effect of nitrite and non-zero charge stripping on the quasi metal free N/C catalyst.

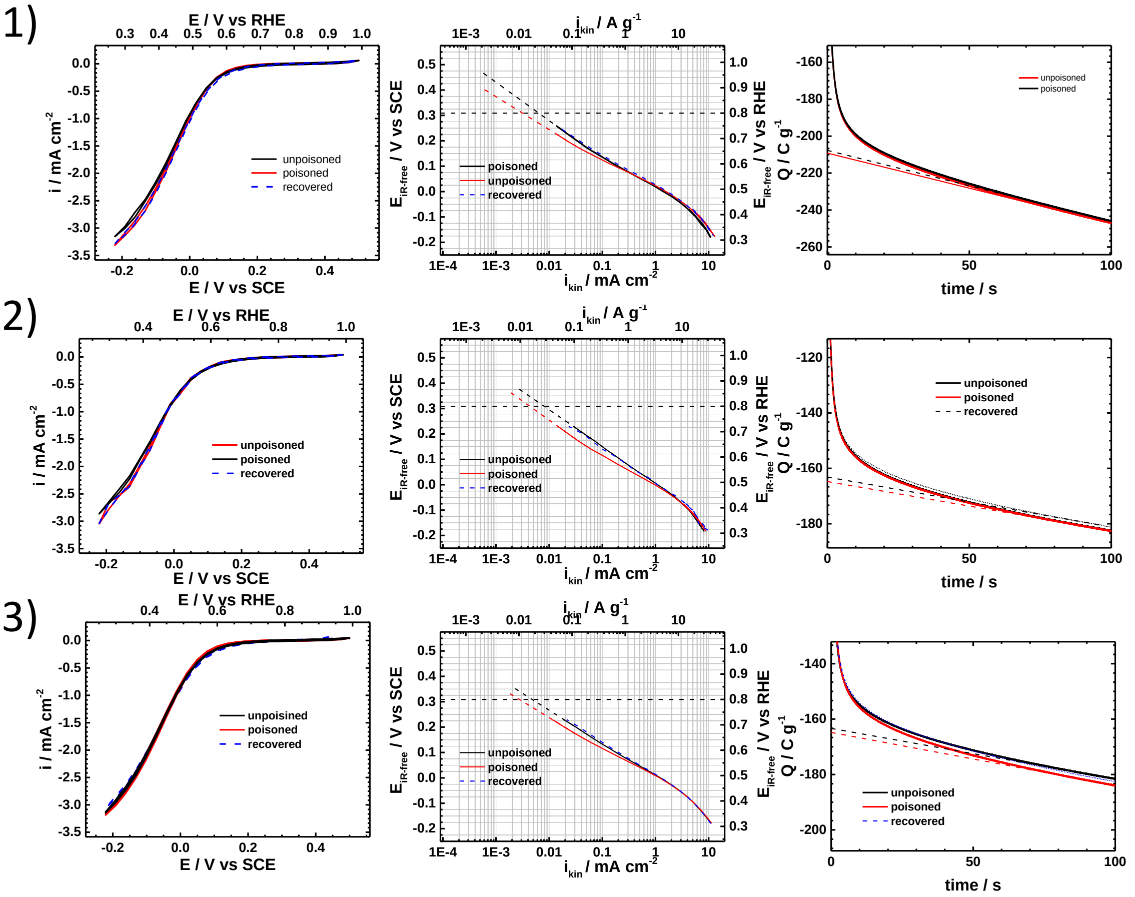

Supplementary Figure 25 ¦ Independent repeat experiments for nitrite poisoning and stripping chronocoulometry on the N/C catalyst. 3 independent repeat measurements for stripping chronocoulometry on the quasi metal free catalyst, to determine the charge that is not associated with the metal centre. (left) ORR measurement in O_2-saturated electrolyte at 5 mV/s. (middle) kinetic current densities as determined from measurement left. Tafel plots extrapolated to 0.8 V vs RHE to allow comparison to the Fe-N/C catalyst. (right) stripping voltammetry in N2-saturated electrolyte at 10 mV/s. 1600 rpm. Loading: 0.27 mg cm^{-2},0.5 M Acetate-buffer, pH 5.2. See Supplementary Tables 3 and 4 for statistical analysis of data in Supplementary Figure 24 and 25. See Supplementary Note 3 for discussion on the effect of nitrite and non-zero charge stripping on the quasi metal free N/C catalyst.

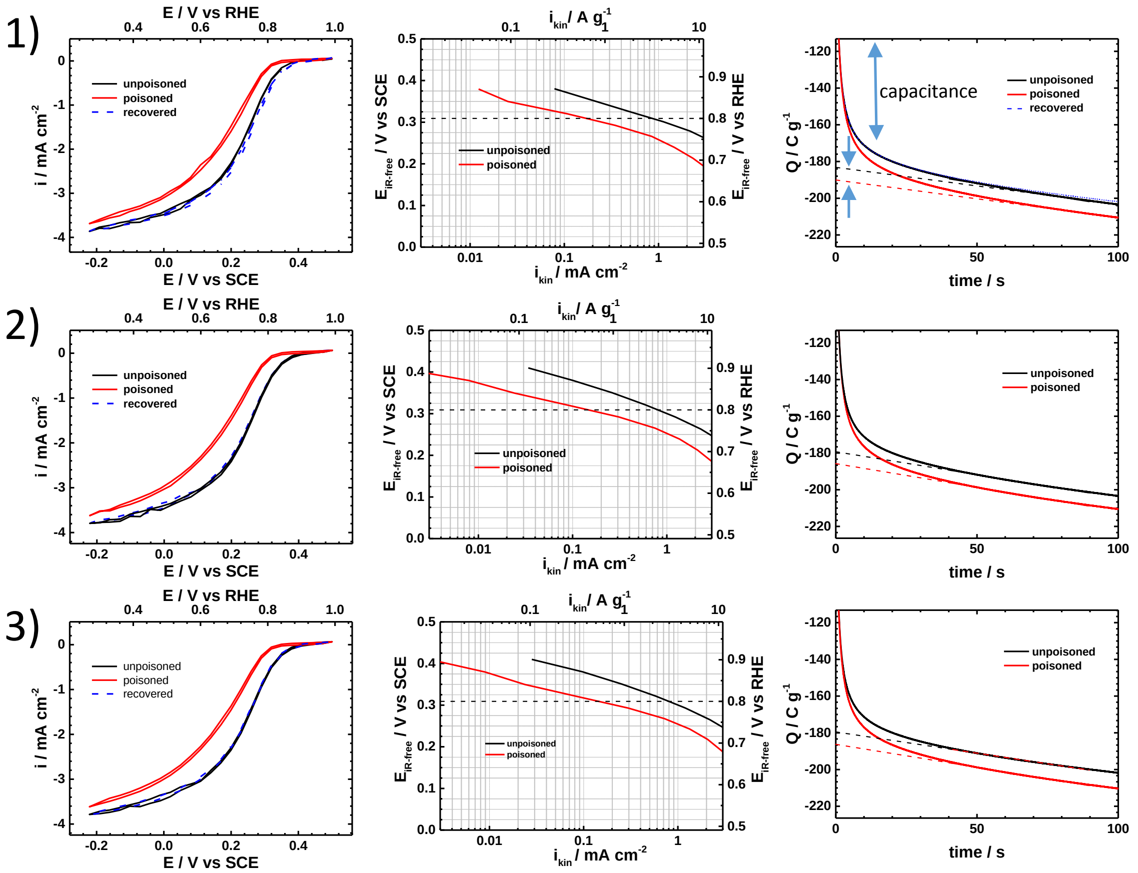

Supplementary Figure 26 ¦ Chronocoulometry repeats compared as alternative to stripping voltammetry. Independent repeat experiments for nitrite poisoning and stripping chronocoulometry on the Fe-N/C catalyst. 3 independent repeat measurements demonstrating the reproducibility of the method. (left) ORR measurement in O_2-saturated electrolyte at 5 mV/s. (middle) kinetic current densities as determined from measurement left. (right) stripping chronocoulometry in N_2-saturated electrolyte at 10 mV/s. 1600 rpm. Loading: 0.27 mg cm^{-2},0.5 M Acetate-buffer, pH 5.2. See Supplementary Table 5 for determined values from each measurement for the metal containing catalyst Fe-N/C.

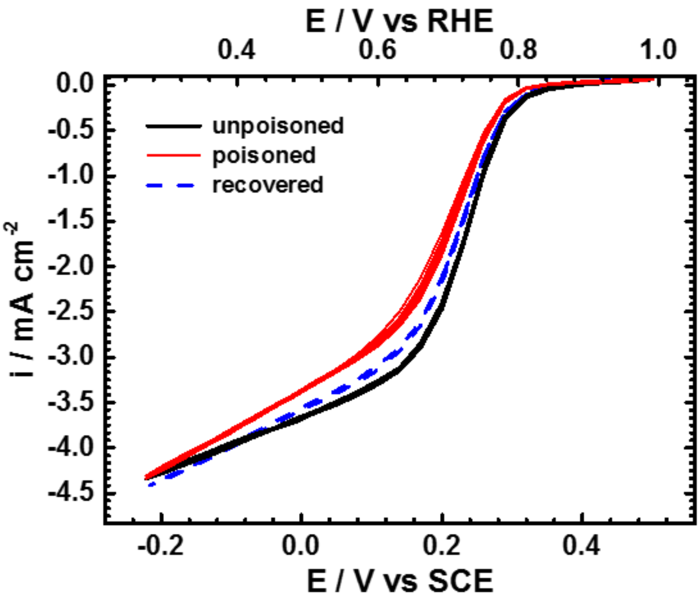

Supplementary Figure 27. Determination of intrinsic activity of FeCo catalyst showing that nitrite stripping method is applicable to other catalysts. ORR on the FeCo catalyst (Supplementary Method 3 for synthesis and Supplementary Table 6 for the differences in the preparation of the Fe-N/C and the FeCo catalysts) before and after the poisoning protocol. Rotating disk electrode measurement of bimetallic FeCo catalyst. Loading: 0.27 mg cm^{-2}, 1600 rpm, O_2-saturated electrolyte, 0.5 M Acetate-buffer, pH 5.2, 5 mV/s. This data shows the ORR activities obtained, following the procedure described in full in supplementary method 2. It can be seen that a similar behaviour to Fe-N/C catalyst can be observed. See Supplementary Method 3 for the synthesis of the FeCo catalyst. Surface area: external: ~395 m^2 g^{-1}; microporous: ~394 m^2 g^{-1}; total: 789 m^2 g^{-1}. MSD, SD, TOF and ORR activity decrease at 0.8 V vs RHE parameters for FeCo compared to Fe-N/C is given in Supplementary Table 6. See supplementary Note 4 for discussion on the hydrogen evolution activity on FeCo affecting the determined nitrite stripping charge and consequently MSD, SD and TOF on this material.

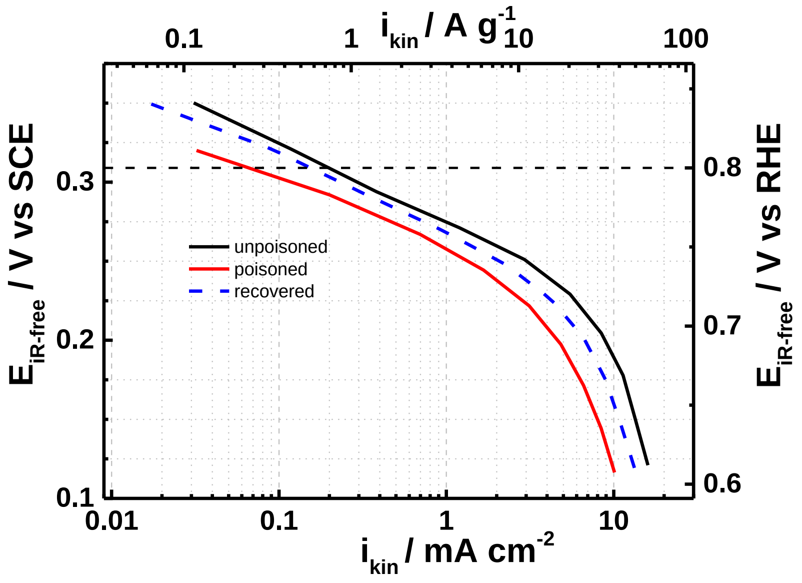

Supplementary Figure 28 ¦ ORR kinetic current density for the ORR on the FeCo catalyst showing that nitrite stripping method is applicable to other catalysts. . It can be seen that in contrast to the Fe-N/C catalyst the FeCo catalyst does not exhibit full recovery. Loading: 0.27 mg cm^{-2}, 1600 rpm, O_2-saturated electrolyte, 0.5 M Acetate-buffer, pH 5.2, 5 mV/s. MSD, SD, TOF and ORR activity decrease at 0.8 V vs RHE parameters for FeCo compared to Fe-N/C is given in Supplementary Table 6. See supplementary Note 4 for discussion on the hydrogen evolution activity on FeCo affecting the determined nitrite stripping charge and consequently MSD, SD and TOF on this material. Supplementary Method 3 for synthesis and Supplementary Table 6 for the differences in the preparation of the Fe-N/C and the FeCo catalysts.

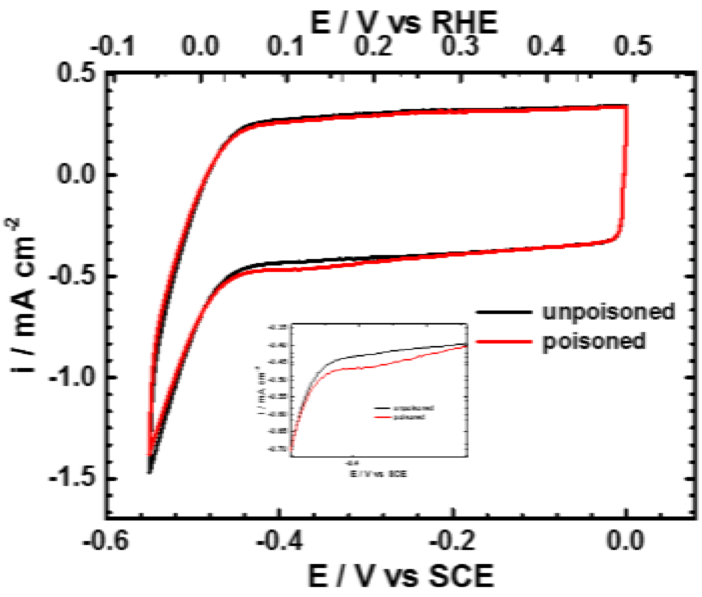

Supplementary Figure 29 ¦ Stripping voltammetry on bimetallic FeCo catalyst showing that nitrite stripping method is applicable to other catalysts.. Loading: 0.27 mg cm^{-2}, 1600 rpm, O_2-saturated electrolyte, 0.5 M Acetate-buffer, pH 5.2, 10 mV/s. It can be seen that there is a clear stripping peak, as also seen for the Fe-N/C catalyst. Unfortunately the hydrogen evolution will become prohibitive at lower potentials and might mask the further observation of the stripping peak. This data shows the associated stripping peak following the procedure described in Supplementary Method 2. MSD, SD, TOF and ORR activity decrease at 0.8 V vs RHE parameters for FeCo compared to Fe-N/C is given in Supplementary Table 7. See supplementary Note 4 for discussion on the hydrogen evolution activity on FeCo affecting the determined nitrite stripping charge and consequently MSD, SD and TOF on this material. Supplementary Method 3 for synthesis and Supplementary Table 6 for the differences in the preparation of the Fe-N/C and the FeCo catalysts) before and after the poisoning protocol.

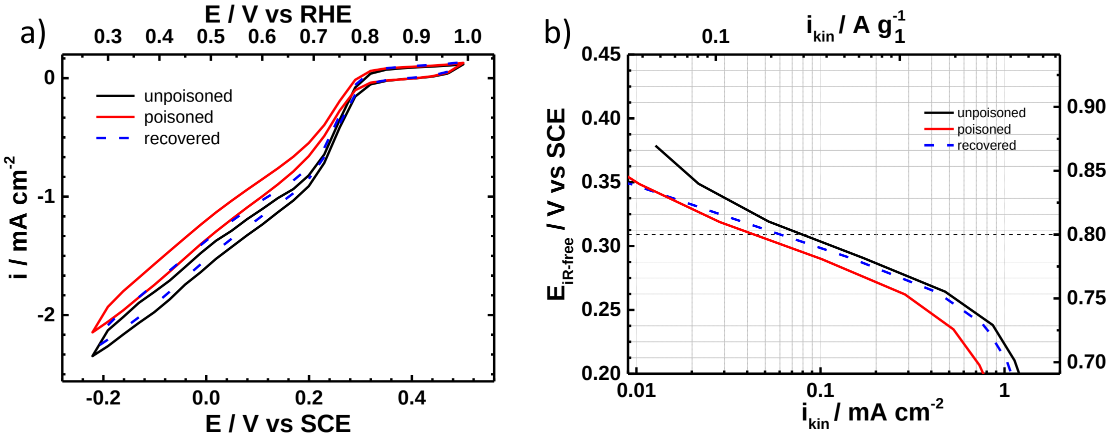

Supplementary Figure 30 ¦ Nitrite poisoning of a Co-N/C catalyst showing that nitrite stripping method is applicable to other catalysts.. a) RDE measurements of the Co-N/C catalyst before and after subjecting it to the poisoning/stripping protocol. b) Kinetic currents. Loading: 0.27 mg cm^{-2}, 1600 rpm, O_2-saturated electrolyte, 0.5 M Acetate-buffer, pH 5.2, 5 mV/s. It can be seen in this data that the activity is lower compared to the Fe-N/C catalyst. Nevertheless, we subjected this catalyst to the same poisoning protocol as shown in the manuscript. It can be seen, that indeed an activity decrease is present, which is less pronounced as for the Fe-N/C catalyst. The activity decrease here is ~45% at 0.8V (RHE). The interaction can either be with Co-N/C site that are nitrite sensitive or with metal free sites. As Co catalyst generally show a more diverse behaviour with a large amount of peroxide produced, these are less interesting to fuel cells.[14] In Supplementary Figure 31, it can be seen that again an excess charge is detected on the poisoned material. We therefore infer that our methodology is applicable to most Fe-N/C catalyst, which represent the most promising of this type of catalysts and are therefore the focus of this study.[14] The methodology might also be useful for some highly active bimetallic catalysts or catalysts based on Co. The nitrite molecular probe might be able to aid in more insight into these catalysts as well. Supplementary Method 3 for synthesis of catalyst.

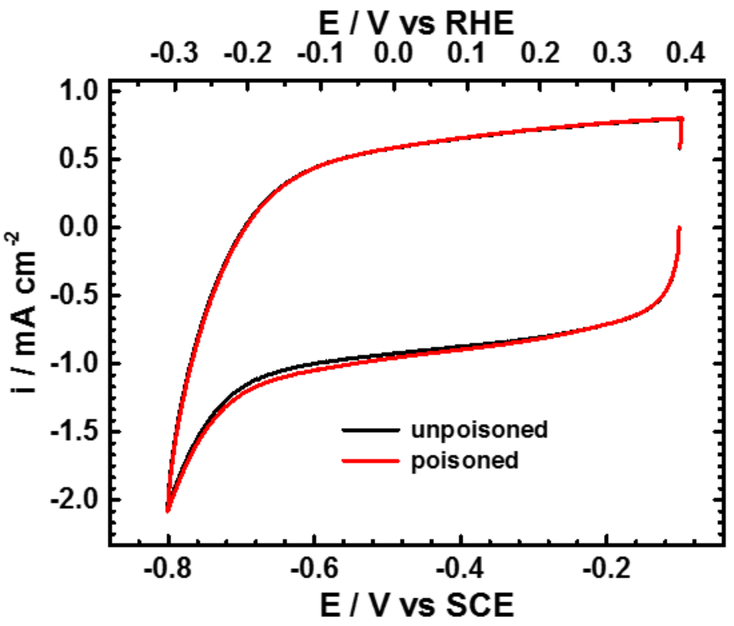

Supplementary Figure 31 ¦ Nitrite stripping of a Co-N/C catalyst showing that nitrite stripping method is applicable to other catalysts.. Stripping voltammetry on the Co-N/C catalyst. Loading: 0.27 mg cm^{-2}, 1600 rpm, O_2-saturated electrolyte, 0.5 M Acetate-buffer, pH 5.2, 10 mV/s. It can be seen that an excess charge is detected on the poisoned material. We therefore infer that our methodology is applicable to most Fe-N/C catalyst, which represent the most promising of this type of catalysts and are therefore the focus of this study.[14] The methodology might also be useful for some highly active bimetallic catalysts or catalysts based on Co. The nitrite molecular probe might be able to aid in more insight into these catalysts as well. Supplementary Method 3 for synthesis of catalyst.

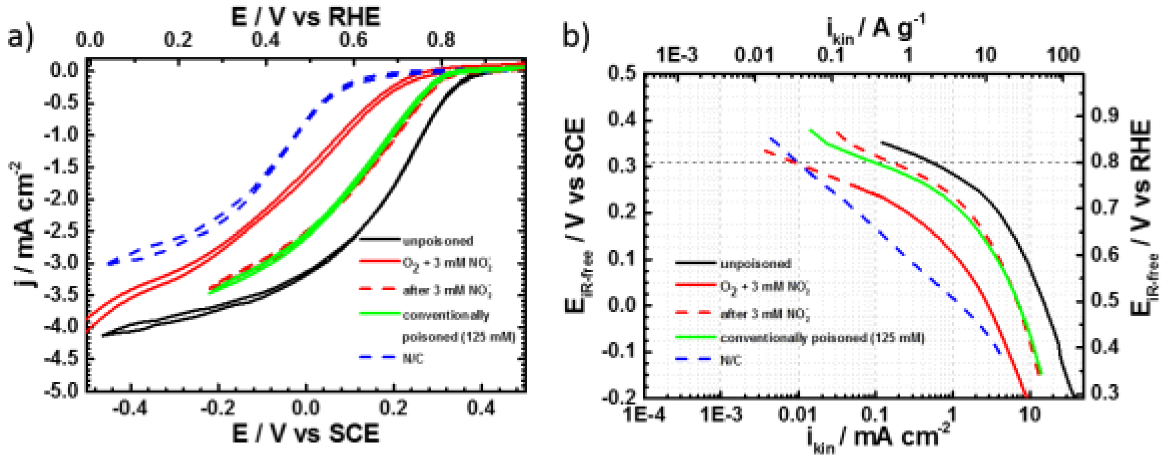

Supplementary Figure 32 ¦ Decrease of the Fe-N/C activity to metal free activity by performing ORR reaction and cycling in nitrite containing solution. a) RDE measurements of the Fe-N/C catalyst under different conditions compared to the N/C catalyst. B) kinetic currents. Loading: 0.27 mg cm^{-2}, 1600 rpm, O_2-saturated electrolyte, 0.5 M Acetate-buffer, pH 5.2, 5 mV/s. It can be clearly seen through these results that in the presence of nitrite, the ORR is clearly inhibited to almost metal free level, while when removing the nitrite from the solution the poisoning is the same (within the accuracy of the measurement) for the 3 mM solution as for the 125 mM solution. This indicates that there are 2 fundamental types of active sites correlated to the iron center. One which interacts with nitrite to bind it and form a stable adduct. This needs to be reductively removed in order to be recovered. The second type however can easily be recovered by washing.

Supplementary Table 1 ¦ BET analysis of Fe-N/C and N/C catalysts for data shown in *Supplementary Figure 6*. The Brunauer–Emmett–Teller (BET) equation was used to extract the surface area. Microporous and external surface area (SA) were determined from the t-plot using the Broekhoff-DeBoer thickness equation. Samples were thoroughly degassed and dried overnight at 300 °C under nitrogen prior to the measurement.

Catalyst	Microporous SA / $m^2\,g^{-1}$	External SA / $m^2\,g^{-1}$	Total SA / $m^2\,g^{-1}$
**Fe-N/C**	421	110	531
**N/C**	550	101	651

Supplementary Table 2 ¦ Mean values and standard deviation for pH, apparent Tafel slopes and current densities at the respective pH for the measurements on Fe-N/C catalyst shown in Figure 2 (main text) from 3 independent runs for each data point. Independent here means that for each run a new catalyst layer from a different newly prepared ink was measured.

**pH**	**Tafel slope / mV dec^{-1}**	**E (i=0.1 mA cm^{-2}) / mV vs SCE**	**E (i=0.1 mA cm^{-2}) / mV vs RHE***
0.30±0.03	83±14	609±12	838±12
1.96±0.01	98±14	528±11	841±11
5.16±0.04	80±12	357±16	848±16
7.00±0.06	97±4.0	230±15	842±15
8.98±0.06	77±14	115±22	839±22

*potential converted to RHE scale as shown in supplementary figure 18 below.

Supplementary Table 3 ¦ Decrease in current density when exposing the quasi metal free catalyst N/C to the poisoning protocol.

Measurement*	i_kin before [mA cm^{-2}]	i_kin after [mA cm^{-2}]	delta_i_kin [mA cm^{-2}]	i_after/i_before
CV1	0.0093	0.0055	0.0038	0.59
CV2	0.0079	0.0049	0.003	0.62
CV3	0.005	0.003	0.002	0.6
CC1	0.0063	0.0031	0.0032	0.49
CC2	0.0079	0.0043	0.0036	0.54
CC3	0.0048	0.0027	0.0021	0.56

*CV means that the stripping voltammetry was used as described in Supplementary Method 2 with the corresponding graphs in Supplementary Figure 25 and CC means that chronocoulometry was used as described in Supplementary Method 2 with the corresponding graphs in Supplementary Figure 26.

Supplementary Table 4 ¦Extracted stripping charge for the metal free catalyst, N/C

Measurement*	Stripping Charge [C g^{-1}]
CV1	2.23
CV2	2.40
CV3	2.11
CC1	1.47
CC2	1.62
CC3	1.45

*CV means that the stripping voltammetry was used as described in Supplementary Method 2 with the corresponding graphs in Supplementary Figure 24 and CC means that chronocoulometry was used as described in Supplementary Method 2 with the corresponding graphs in Supplementary Figure 25.

Supplementary Table 5 ¦ Extracted stripping charge and ORR kinetic current data for the Fe-N/C catalyst and MSD, TOF, SD and iron utilisation on of the Fe-N/C catalysts as per the nitrite stripping method. Determined values from each measurement for the metal containing catalyst Fe-N/C from data in Supplementary Figure 23 and equations 5 to 7 in the main text.

Measurement *	i_kin before [mA cm^{-2}]	i_kin after [mA cm^{-2}]	delta_i_kin [mA cm^{-2}]	i_after/i_before	Stripping Charge [mC]
**CV1**	0.3870	0.0855	0.3015	0.2209	0.2550
**CV2**	0.5080	0.0910	0.4170	0.1791	0.2910
**CV3**	0.5170	0.1060	0.4110	0.2050	0.2670
**CC1**	0.8110	0.1680	0.6430	0.2072	0.3470
**CC2**	0.7770	0.1460	0.6310	0.1879	0.3500
**CC3**	0.7450	0.1410	0.6040	0.1893	0.3600
**AVERAGE**	0.6242	0.1229	0.5013	0.1982	0.3117
**STDEV**	0.1756	0.0335	0.1433	0.0154	0.0462

Measurement *	MSD [mmol g^-1]	TOF [s^-1]	SD_external [nm^-2]	SD_total [nm^-2]	Iron utilisation [%]
**CV1**	0.0100	1.1605	0.0601	0.0124	3.7127
**CV2**	0.0114	1.4065	0.0686	0.0142	4.2368
**CV3**	0.0104	1.5108	0.0629	0.0130	3.8874
**CC1**	0.0136	1.8187	0.0818	0.0169	5.0522
**CC2**	0.0137	1.7695	0.0825	0.0171	5.0958
**CC3**	0.0141	1.6467	0.0848	0.0176	5.2414
**AVERAGE**	0.0122	1.5521	0.0734	0.0152	4.5377
**STDEV**	0.0018	0.2464	0.0109	0.0023	0.6731

*CV means that the stripping voltammetry was used as described in Supplementary Method 2.2 with the corresponding graphs in Supplementary Figure 23 and CC means that chronocoulometry was used as described in Supplementary Method 2.

Supplementary Table 6 ¦ Differences in the preparation of the Fe-N/C and FeCo catalysts. Highlighting the differences in the preparation route to obtain the two different catalysts. Differences in the preparation of the Fe-N/C and FeCo catalysts:

	**Fe-N/C**	**FeCo**
1	Literature method[10]	Literature method[9]
2	No carbon support. Forms the conductive structure from precursor	Carbon support used into which the active sites are somehow incorporated
3	Aromatic nitrogen source (1,5 Diaminonaphthalene) with two nitrogen atoms per molecule. Is polymerised before pyrolysis and presumably strongly interacts with the metal centre	Molecular non aromatic nitrogen source (N-ethylamine) with only one nitrogen atom per molecule. Is added to the mixture and presumably reacts with the oxidised carbon structure[9]
4	Monometallic (iron)	Bimetallic (iron and cobalt)
5	Inactive towards hydrogen evolution	Active towards hydrogen evolution
6	Second heat treatment	No second heat treatment
7	High micropore area and low external surface area (~110 m^2 g^{-1})	Medium micropore area and high external surface area (~395 m^2 g^{-1})
8	No evidence for particulate phases i.e. Fe_3O_4[10]	Evidence for Fe_3O_4 and Co_3O_4 phases[9]

It can be clearly seen that the preparation routes for the catalysts lie on the two extremes of the spectrum for synthesis procedures described in literature and the materials exhibit significant differences in their behaviour.[11]

Supplementary Table 7 ¦ Demonstrating the differences in the crucial catalyst parameters of Fe-N/C versus FeCo, showing that interesting conclusions can be drawn with the help of this method.

Catalyst	Q / C g^{-1}	MSD / mmol g^{-1}	SD_external / nm^{-2}	SD_total / nm^{-2}	TOF @0.8V (RHE) / electrons $site^{-1}$ s^{-1}	Activity decrease @ 0.8V(RHE) / %
**FeCo**	**~3.89**	**~0.008**	**~0.05**	**~0.01**	**~0.43**	**~63**
**Fe-N/C**	**~5.87**	**~0.012**	**~0.07**	**~0.02**	**~1.55**	**~80**

Supplementary Note 1

The use of pH 5.2 for the stripping experiments

Supplementary Figure 13 compares the poisoning/stripping behaviour in the acidic electrolyte to the behaviour at pH5.2, when the concentration of the poison used was 12.2M. The same procedure has been used as described in the main text. The difference for the data shown in Supplementary Figure 13 (a and b) is that all steps in 0.5M acetate buffer have been replaced by 0.5M H_2SO_4 while for the data shown in Supplementary Figure 13 (c and d) the poisoning step has been conducted in saturated (~12.2M) $NaNO_2$ solution instead of the usually used 0.125M. This clearly demonstrates that this slight difference in the behaviour at different pH is not caused by a change in the active site or the mechanism, but by the presence of NO and/or NO_2 caused by the decomposition of nitrite at low pH or the presence in the poisoning solution at high concentrations of nitrite. We believe that this presence causes interference with the stripping protocol. Therefore using pH 5.2 is directly applicable.

In Supplementary Figure 10 it can be seen that when subjecting the catalyst to the poison and stripping protocol in acid, the activity decrease is comparable to the activity decrease at pH 5.2. In Supplementary Figure 11 it can also be seen that a clear stripping peak is present and a recovery is observed. However, we found it more suitable to perform this study at pH 5.2 and reasons are outlined below:

i) Nitrous acid decomposes to releases a complex mixture of molecules due to $2HNO_2 \leftrightarrows NO_2 + NO + H_2O$ where the pK_A of HNO_2 is 3.4. At pH 5.2, the equilibrium concentration of HNO_2 in a 0.125 M nitrite solution is 2 mM (i.e. 1.5% exists as nitrous acid). At pH 0,3, 99.96% of the nitrite exists as nitrous acid, and the rate of the disproportionation reaction is very fast (4000x faster assuming the rds is the initial reaction between the two HNO_2 molecules).
ii) NO_2 and NO react quite differently with iron and produce different complexes, some of which (e.g. nitrosyl complexes) are very stable.
iii) It can be seen that the recovery is not 100%. This is attributed to the formation of the more aggressive NO and NO_2 species (c.f. Supplementary Figure 11 b which compares the same result but in pH 5.2 buffer – the recovered scan exactly overlays the initial scan). This is further confirmed by reproducing the acidic behaviour when a higher nitrite concentration is used as a poison.
iv) It can also be seen that the baselines for the unpoisoned and recovered scans do not align perfectly as is the case at pH 5.2
v) At low pH, it is impossible to correlate the stripping peak with the reduction of nitrite, as this molecule is not stable under these conditions, complicating the analysis
vi) The fact that the stripping peak, together with the fact that the rate is linearly dependent on pH in the range of pH 0 to pH 9 strongly suggests that the same active sites are present at both pH values. Therefore, measurements at pH5.2 are directly transferable to lower pH values
vii) The uncertainty we found at pH 0.3, which we define by the discrepancy between unpoisoned stripping trace and recovered trace, is significantly larger at pH 0.3 than at pH 5.2, making the quantification challenging.

For these reasons we choose the pH 5.2 in our study. Nevertheless, a lower pH might also be chosen if a suitable error analysis is performed or suitable steps are undertaken in order to ensure a correct baseline.

Supplementary Note 2

The choice of 0.125 M as the nitrite concentration

In order to improve the understanding of the nitrite interaction with the different types of active sites and to validate the here presented methodology, the concentration of the nitrite containing poisoning solution was varied, while all other parameters where kept constant. Supplementary Figure 19(a) shows the ORR performance before and after subjecting the catalyst layer to these different concentrations. Supplementary Figure 20 shows the respective kinetic currents. The nitrite concentration has been varied over 8 orders of magnitude, in order to cover a wide spectrum of interactions. Supplementary Figure 20 shows decrease in activity plotted versus the logarithm of nitrite concentration. It can be clearly seen that at very low concentrations, the decrease in kinetic current is dependent on the concentration of nitrite. This effect persists to approximately millimolar level. Going to higher concentrations, a plateau is reached which extends over at least 2 orders of magnitude concentration difference. When increasing the concentration further (saturated solution), there is again a slight further decrease in activity. The associated correlation of stripping charge with the nitrite concentration is shown in Supplementary Figure 21. It can be seen that the same behaviour is observed and a plateau is reached which extends over 2 orders of magnitude in nitrite concentration. After exposing the catalyst to the saturated nitrite solution, there is a significant increase in stripping charge. Supplementary Figure 21(a) shows the correlation of stripping charge with activity decrease. It can be seen that over a wide range (6 orders of magnitude in poison concentration) there is a good correlation of activity decrease with stripping charge. Only when exposed to the saturated solution, the charge deviates from this direct correlation. This might possibly be reasoned in terms of a behaviour that is analogous to an isothermal adsorption of a species with a saturation process and a second mode of adsorption thereafter i.e. BET adsorption. Figure 21(b) tries to explain this behaviour. We include 2 different types of metal based active sites into this hypothesis, as also suggested by our results. We propose a strongly interacting type A and a weaker interacting type B. At low concentrations not all active sites of type A are covered (Supplementary Figure 8.3a) i.e. from 125 nM to 12.5 µM. Only at sufficiently high poisoning concentration, all active sites of type A are covered (Supplementary Figure 21(b). This is where the plateau is reached and where the concentration of nitrite used in this study was chosen. Increasing the concentration further results in the unspecific adsorption of nitrite onto the catalyst surface (Supplementary Figure 21(c). The further decrease in activity could either be due to blocking of the active site or other undefined effects. This is also where a significant increase in stripping charge is observed. Nevertheless, the here conducted study clearly shows that the activity decrease is correlated with the stripping charge, making our methodology suitable to determine catalyst parameters. It furthermore confirms that the concentration used to poison the catalyst in this study is chosen appropriately, as it lies in the region where only specific adsorption to active sites is likely.

Supplementary Note 3

The effect of nitrite on to the quasi metal free N/C catalyst: While the effect on the metal free catalyst is small, it is still significant. From the extrapolation of the Tafel plot to 0.8 V vs RHE it can be seen that upon nitrite exposure there is an activity decrease of 43±4 % as compared to ~80% for the Fe-N/C catalyst. This is most likely due to the low but still finite residual metal content of ~60ppm. It can also be seen in the Tafel plots in Supplementary Figure 24 and 25 (middle) that the poisoning effect is only present at higher potentials. Nitrite treatment might therefore also reveal metal contributions in presumably metal free catalysts.

The non-zero stripping charge on the N/C catalyst: Ideally the quasi metal free catalyst should exhibit a zero stripping charge. However. Even on the metal free catalyst, a significant excess charge of **1.9±0.4 C g^{-1}** as compared to **6.0±1 C g^{-1}** is measured upon nitrite stripping. This is 32% of the metal associated charge. This means in the worst case the calculated turnover frequency is underestimated by ~30%. Therefore the determined TOF of 1.60 s^{-1} might be as high as 2.35 s^{-1}. Nevertheless, this fact does not reduce the significance of the here demonstrated method, as activity trends can be followed and the here presented direct correlation can be used to unravel activity descriptors.

The stripping charge might be attributed to different sources:

1.) The residual metal content will be covered by nitrite and hence contribute to the stripping charge on the N/C catalyst. However this amount should be rather small. If all the metal (60ppm) in the catalyst was on the surface and would contribute to the stripping charge, the maximum charge to be obtained would be ~0.53 C g^{-1}. Therefore, only a fraction of the observed charge is to be attributed to the residual metal.
2.) Other metal free active sites might also interact with nitrite, however much weaker.
3.) Even though care was taken to wash off unspecifically adsorbed nitrite molecules, there might still be a residual amount that might be electrochemically reduced.
4.) A chemical reaction between the nitrite containing solution and the carbon surface of the catalyst increases slightly the capacitance and therefore the baseline in the potential region of interest is slightly shifted.
5.) A general uncertainty in the capacitance due to the transfer of the catalyst layer between solutions can contribute to a parasitic charge, which might lead to a systematic error.
6.) A systematic error caused by other unknown sources.

Supplementary Note 4

Difficulty in analysis of Co-N/C catalysts

It can be seen that the difficulty in evaluating the Co containing catalyst FeCo is that it exhibits a significant hydrogen evolution activity. This will mask the stripping peak and make the use of this method challenging. Nevertheless, a stripping peak with associated recovered activity can be identified and analysed. The extracted parameters for FeCo are given in Supplementary Table 6 and compared to the Fe-N/C catalyst. The hydrogen evolution activity is presumably caused by the Co content, as there are several reports in literature for cobalt containing hydrogen evolution catalysts, either atomic or as chalcogel.[12, 13] However, to the best of our knowledge, there is no report of highly active iron-only hydrogen evolution catalysts with similar structure to the Fe-N_x type catalyst investigated here. We therefore infer that the stripping method should work excellently for most Fe-N/C iron-only catalysts with Fe-N_x sites and can give useful information for other non-precious metal catalysts. Interestingly, the activity decrease of the catalyst FeCo is relatively lower (17% difference) than the Fe-N/C catalyst (see Supplementary Table 6). This suggests that while a similar set of active sites is present in both catalysts, the catalyst FeCo might also contain a second set of active sites which is not affected by nitrite. This could possibly be attributed to the particulate phases or the cobalt content of the FeCo catalyst.

Supplementary Method 1

Preparation and measurement of samples for Total Reflection X-ray Fluorescence (TXRF)

TXRF was conducted on a Bruker S2 Picofox. Samples were prepared from a suspension of 10 mg catalyst in 1 mL H_2O (MiliQ 18.2 MΩ·cm), which contained 1 wt% Triton X-100 (Sigma Aldrich) as surfactant, 0.2 wt% polyvinylalcohol (Mowiol® 4-88, Sigma-Aldrich) as binder and 100 µg Ga, as internal standard (from 1 g/L Standard Solution, TraceCert®, Sigma-Aldrich). 10 µL were deposited onto a quartz glass sample carrier and dried at room temperature in a laminar flow hood to give a homogenous thin film. 3 independent repeat measurements from different suspensions were conducted for each catalyst. The iron content was determined to be 1.5±0.2 wt% in the Fe-N/C catalyst and 60±20 ppm in the N/C catalyst. For Fe, the characteristic 6.4 keV K α1 emission line was used for calibration and quantification.[5] Other detected trace elements (Zn and Cu) were below the 100 ppm threshold which was suggested as value to differentiate between metal free and metal containing catalysts.[6]

Supplementary Method 2

Electrochemical Cleaning Protocol

In order to obtain a non-changing oxygen reduction performance and non-changing cyclic voltammograms under nitrogen for the duration of the experiment, the catalyst layer has to be conditioned. Therefore extensive cycling was performed in order to remove entrapped gas bubbles within the catalyst layer, making the layer hydrophilic and allow a complete wetting. The procedure consisted of extensive cycling, alternating between N_2-saturated electrolyte at 100 mV/s (20 cycles) and 10 mV/s (10 cycles) and O_2¬-saturated electrolyte at 5 mV/s (6 cylces), in the potential window 1.05 - -0.4 V vs RHE. This was repeated until stable non changing oxygen reduction performance and cyclic voltammograms under nitrogen were achieved (ca. 3 - 4 times).

2.1 Protocol for determination of adsorbed Nitrite using cyclic voltammetry

After the typical break-in procedure and background scan, a) Record the unpoisoned ORR performance in O_2-saturated electrolyte, 1.0 - 0.3 V vs RHE, 5 mV/s b) Record a pre-baseline cyclic voltammogramm (CV) in N_2-saturated electrolyte 1.0 – 0.3 V vs RHE, 10 mV/s. This is necessary, in order remove any residual dissolved oxygen which might falsify the results. It was furthermore found that this step increases the accuracy of the baseline during the stripping scan in the region of interest. c) Record a baseline CV in N_2-saturated electrolyte 0.4 - -0.3V vs RHE, 10 mV/s d) dip RDE into 125 mM $NaNO_2$ solution for 5 minutes at open circuit potential (OCP), 300 rpm e) wash electrode in DI water for 1 minute at OCP, 300 rpm f) wash electrode in electrolyte solution for 5 minutes at OCP, 300 rpm g) wash electrode in DI water for 1 minute at OCP, 300 rpm; the washing steps were found to be necessary in order to remove unspecifically adsorbed nitrite and to avoid nitrite contamination of the electrolyte. h) record ORR of poisoned electrode in O_2-saturated electrolyte, 1.0 - 0.3 V vs RHE, 5 mV/s i) record pre-baseline CV of poisoned electrode in N_2-saturated electrolyte, 1.0 - 0.3 V vs RHE, 10 mV/s j) record stripping CV in N_2-saturated electrolyte, 0.4 - -0.3 V vs RHE, 10 mV/s . Following this step, steps (h)-(j) were repeated to confirm recovery of ORR performance and baseline CV.

2.2 Protocol for determination of adsorbed Nitrite using Chronoamperometry

When chronocoulometry is used instead of stripping voltammetry, the ORR steps remain the same. The pre-baseline CV is replaced with 240s of chronoamperometry @0.3 V vs RHE (stepped from 0.79 V vs RHE 5s) in N_2-saturated electrolyte at a rotation of 1600 rpm, in order to remove any dissolved oxygen or unspecifically adsorbed nitrite. Then the baseline step of the unpoisoned catalyst and stripping voltammetry step of the poisoned catalyst are replaced with a chronocoulmetry step under nitrogen at a rotation rate of 1600 rpm. The chronocoulometry step was performed by first equilibrating the surface @0.79 V vs RHE for 5s and then stepping to -0.29 V vs RHE to record the charge passed versus time for 240 s (Figure S 10.1). In order to control potential and measure charge an Autolab PGSTAT 20 potentiostat equipped with an integrator module was used. The stripping charge can then be extracted by plotting the integrated charge versus time and extrapolating the linear region to t=0. The difference in the intercept of the unpoisoned to the poisoned catalyst is the charge associated with nitrite stripping. The small steady increase in charge in the linear region is due to the non-zero constant current associated with hydrogen evolution at this potential (-0.1 V vs RHE) and rotation rate (1600 rpm).

Supplementary Method 3

Synthesis of FeCo catalyst:

The catalyst denoted FeCo was synthesized following a published method that was slightly modified.[9] In a typical route, 0.5 g of carbon black (Ketjenblack® EC-600JD) was refluxed in 200 mL of a 1.0 mol dm^{-3} HCl solution at 80 °C for 8 h to remove trace metals from the carbon black. Subsequently, this was vacuum filtrated, washed with plenty of ultrapure water (18.2 MΩ cm) and dried at 80 ^{o}C for 10 h. 200 mg of this pre-treated carbon black was weighted and dispersed in approximately 70 mL of absolute ethanol in an ultrasonication bath. A separate beaker was used to dissolve, in 30 mL of absolute ethanol (VWR), 0.0058 g of $FeCl_3$ (Sigma-Aldrich), 0.0063 g of $Co(NO_3)_2$ (Sigma-Aldrich) along with 2 mL of N-ethylamine (Sigma Aldrich). This solution was added to the carbon black suspension and let to reflux for 8 h at 80 °C in a water bath. Subsequently, the resulting solids were filtered and washed with ultrapure water and dried in an oven at 100 °C for 8 h. The dried powders were heat-treated in a tubular oven at 900 °C under nitrogen atmosphere for 1 h after ramping the temperature from room temperature at 20 °C min^{-1}. Finally, the catalyst was refluxed in 100 mL of 0.5 mol dm^{-3} H_2SO_4 at 80°C for 24 h to remove soluble metal phases. This suspension was vacuum filtrated and washed with excess ultrapure water and dried at 80 ^{o}C for 10 h. BET analysis of the resultant material gave the following results: Surface area: external: ~395 m^2 g^{-1}; microporous: ~394 m^2 g^{-1}; total: 789 m^2 g^{-1}.

Synthesis of Co-N/C catalyst

The synthesis procedure for the Co-N/C catalyst is the same as for the FeCo catalyst above, with the only difference of replacing $FeCl_2$ with $CoCl_2$*($6H_2O$) (Sigma-Aldrich, 99.9%).

Supplementary References:

1. Z.-S. Wu, S. Yang, Y. Sun, K. Parvez, X. Feng and K. Müllen, *Journal of the American Chemical Society*, 2012, **134**, 9082-9085.
2. H. Jiang, Y. Yao, Y. Zhu, Y. Liu, Y. Su, X. Yang and C. Li, *ACS Applied Materials & Interfaces*, 2015, **7**, 21511-21520.
3. J. Wang, G. Wang, S. Miao, X. Jiang, J. Li and X. Bao, *Carbon*, 2014, **75**, 381-389.
4. T. Denaro, V. Baglio, M. Girolamo, V. Antonucci, A. S. Arico', F. Matteucci and R. Ornelas, *Journal of Applied Electrochemistry*, 2009, **39**, 2173-2179.
5. G. Hölzer, M. Fritsch, M. Deutsch, J. Härtwig and E. Förster, *Physical Review A*, 1997, **56**, 4554-4568.
6. J. Masa, W. Xia, M. Muhler and W. Schuhmann, *Angewandte Chemie International Edition*, 2015, **54**, 10102-10120.
7. E. J. Biddinger, D. v. Deak, D. Singh, H. Marsh, B. Tan, D. S. Knapke and U. S. Ozkan, *Journal of The Electrochemical Society*, 2011, **158**, B402-B409.
8. N. Ramaswamy, U. Tylus, Q. Jia and S. Mukerjee, *Journal of the American Chemical Society*, 2013, **135**, 15443-15449.
9. J.-Y. Choi, R. S. Hsu and Z. Chen, *The Journal of Physical Chemistry C*, 2010, **114**, 8048-8053.
10. D. Malko, T. Lopes, E. Symianakis and A. R. Kucernak, *Journal of Materials Chemistry A*, 2015, **4**, 142-152.
11. F. Jaouen, E. Proietti, M. Lefèvre, R. Chenitz, J.-P. Dodelet, G. Wu, H. T. Chung, C. M. Johnston and P. Zelenay, *Energy & Environmental Science*, 2010, **4**, 114-130.
12. H. Fei, J. Dong, M. J. Arellano-Jiménez, G. Ye, N. Dong Kim, E. L. G. Samuel, Z. Peng, Z. Zhu, F. Qin, J. Bao, M. J. Yacaman, P. M. Ajayan, D. Chen and J. M. Tour, *Nature Communications*, 2015, **6**, 8668.
13. J. Staszak-Jirkovský, C. D. Malliakas, P. P. Lopes, N. Danilovic, S. S. Kota, K.-C. Chang, B. Genorio, D. Strmcnik, V. R. Stamenkovic, M. G. Kanatzidis and N. M. Markovic, *Nature Materials*, 2016, **15**, 197-203.
14. M. Shao, Q. Chang, J.-P. Dodelet and R. Chenitz, *Chemical Reviews*, 2016, **116**, 3594-3657.
